# Tumor–immune metabolic tug-of-war: from immune escape to targeting metabolic rewiring in cancer therapy

**DOI:** 10.3389/fcell.2026.1823145

**Published:** 2026-06-05

**Authors:** Beatrice Foglia, Jessica Nurcis, Marta Signorini, Stefania Cannito

**Affiliations:** Unit of Experimental Medicine and Clinical Pathology, Department of Clinical and Biological Sciences, University of Torino, Torino, Italy

**Keywords:** immune targeted therapies, immunity, metabolic reprogramming, oncometabolites, tumor microenvironment, tumor–stroma interaction

## Abstract

Cancer remains one of the leading causes of death worldwide, with nearly 20 million new cases and 9.7 million deaths reported in 2022. Projections estimate that annual incidence may increase to 35 million by 2050, underscoring cancer as both a biomedical challenge and a global socioeconomic burden. A key hallmark of malignancy that sustains this progression is metabolic reprogramming. Within the nutrient- and oxygen-deprived tumor microenvironment, cancer cells rewire their metabolism to generate the energy and biosynthetic intermediates necessary for unchecked proliferation, invasion, and metastasis. This metabolic adaptation, however, extends beyond tumor growth. By competing for critical nutrients such as glucose and glutamine, cancer cells establish a metabolic tug-of-war with immune cells, impairing their activation and antitumor functions. In addition, cancer cells produce oncometabolites that act as signaling molecules, fostering immune suppression. Together, nutrient competition and oncometabolite signaling contribute to immune evasion and therapeutic resistance. In this review, we examine the dynamic metabolic dialogue between tumors and the immune system, focusing on how cancer cells induce immune metabolic rewiring, how immune cells respond or succumb to these changes, and the therapeutic opportunities emerging from targeting metabolic vulnerabilities on both sides of the tug-of-war.

## 1 Introduction

In 2022, the global cancer burden was estimated at nearly 20 million new cases and approximately 9.7 million deaths, with approximately 20% of the population expected to develop cancer over a lifetime and mortality risks of roughly 11% for men and 8% for women. Lung, breast, colorectal, prostate, and stomach cancers together accounted for a large proportion of both incidence and mortality worldwide. These results reflect substantial geographic and socioeconomic disparities in both incidence and outcomes, with higher rates in high-income countries and disproportionately high mortality in resource-limited regions (Bray et al., 2024). By 2025, global estimates indicate more than ∼20 million new cancer cases and over 10.3 million deaths annually, with five-year prevalence exceeding 50 million people, underscoring cancer’s continued status as a leading cause of illness and death globally and persistent inequalities in burden and resources for care (American Association for Cancer Research, 2025). Looking ahead to 2050, projections based on demographic trends and GLOBOCAN modeling suggest the global cancer burden will continue to increase sharply, with an estimated 35.3 million new cases (≈+76.6%) and 18.5 million deaths (≈+89.7%) compared with 2022 (Bizuayehu et al., 2024). These increases are expected to be driven by population aging and growth, alongside persistent risk exposures and unequal access to prevention and care across countries. This trend further emphasizes the urgent need to deepen our understanding of the biological mechanisms underlying tumor development and progression (American Cancer Society, 2024).

In this context, addressing cancer as a global health challenge requires not only epidemiological surveillance but also a deeper understanding of the fundamental biological processes that sustain tumor growth and progression. Increasing attention has been directed toward the biological hallmarks that enable tumors to adapt, survive, and progress, among which metabolic reprogramming has emerged as a central and unifying mechanism. Cancer is now recognized as a disease driven not only by genetic and epigenetic alterations but also by profound metabolic rewiring, which has evolved from an emerging hallmark to a core and enabling feature of malignancy (Hanahan, 2022).

Since Warburg’s seminal observation that cancer cells exhibit elevated glucose uptake, the field of cancer metabolism has shown that malignant transformation and tumor progression involve a cell-wide metabolic rewiring extending far beyond aerobic glycolysis. This rewiring is not limited to increased glucose consumption but involves coordinated alterations in multiple interconnected pathways, including sustained aerobic glycolysis, mitochondrial remodeling, and modulation of oxidative phosphorylation (OXPHOS), along with activation of key nutrient- and oxygen-sensing signaling axes such as PI3K–AKT–mTOR and HIF-1α. These pathways integrate environmental cues with cellular metabolic demands, enabling tumor cells to dynamically adapt to hypoxia, nutrient deprivation, and immune pressure. Cancer cells commonly shift glucose and nutrient metabolism (e.g., enhanced glycolysis and altered lipid and amino acid use) to fuel biosynthesis and redox balance, contributing to heterogeneity, therapy resistance, and adaptation in nutrient-poor microenvironments (Hanahan, 2022; Pavlova and Thompson, 2016). Importantly, these metabolic programs are not merely bioenergetic adaptations but actively shape immune evasion. Enhanced glycolysis and lactate production acidify the tumor microenvironment, suppressing effector T and NK cell function, while competition for key nutrients such as glucose and glutamine limits immune cell activation and persistence. In parallel, mitochondrial and signaling rewiring through the mTOR and HIF-1α pathways modulates the differentiation and function of myeloid and lymphoid populations, promoting immunosuppressive phenotypes such as regulatory T cells, tumor-associated macrophages, and myeloid-derived suppressor cells (Pavlova and Thompson, 2016; Xia et al., 2021; De Martino et al., 2024).

These widespread and often subtle metabolic adaptations can be conceptualized as a hidden driving force of tumor progression, shaping tumor behavior in complex, not always immediately observable ways (Chiu et al., 2024). Crucially, accumulating evidence indicates that cancer-associated metabolic rewiring also plays a decisive role in evading anticancer immunosurveillance, a major selective pressure during host-tumor co-evolution. Moreover, this reprogramming not only sustains tumorigenesis but also profoundly influences the immune landscape of the tumor microenvironment (TME): metabolic competition and secreted metabolites can suppress antitumor immune responses and shape immune cell differentiation and function, presenting both challenges and opportunities for therapy (De Martino et al., 2024; Xia et al., 2021). These findings reinforce the concept that oncogenesis is not purely a cancer cell-intrinsic process but also depends on cancer cell-extrinsic metabolic interactions that enable immune escape.

Considering the rising global cancer burden and increasing therapeutic resistance, a comprehensive understanding of tumor–immune metabolic crosstalk is essential. In this review, we aim to provide an integrated overview of the metabolic interactions between cancer and immune cells within the tumor microenvironment, highlighting key pathways, their impact on antitumor immunity, and their potential as targets for therapeutic intervention.

## 2 Hooked on sugar, forged in hypoxia: the glycolytic-mitochondrial engine of cancer rewiring

Cancer cells undergo profound metabolic rewiring to sustain proliferation, survival, and immune evasion within the TME. A hallmark of this transformation is enhanced aerobic glycolysis, known as the Warburg effect, characterized by increased glucose uptake and preferential conversion of pyruvate to lactate even in the presence of oxygen (De Martino et al., 2024; Kao et al., 2022; Zhang H. et al., 2024). Tumor cells exploit this metabolic shift to sustain rapid proliferation, anabolic growth, and redox balance while regenerating NAD^+^ through lactate dehydrogenase A (LDHA)-mediated conversion of pyruvate to lactate (Comandatore et al., 2022). This glycolytic switch progressively depletes interstitial glucose and drives the accumulation of lactate and protons, generating an acidic, nutrient-poor, and hypoxic microenvironment in which tumor cells maintain robust proliferation, whereas immune cells experience profound bioenergetic stress (Poznanski et al., 2021; Lian et al., 2022).

Tumor glycolysis is sustained by oncogenic signaling pathways that converge on key metabolic regulators, including phosphatidylinositol 3-kinase (PI3K)–AKT serine/threonine kinase (AKT)–mTOR, HIF-1α, and c-Myc. These pathways upregulate glucose transporters (GLUT1 and GLUT3) and glycolytic enzymes such as hexokinase 2 (HK2), pyruvate kinase M1/2 (PKM), and LDHA, reinforcing high glycolytic flux (Gabrilovich, 2017; Li et al., 2017; Hu et al., 2020; Wang Y. et al., 2020). Elevated serum LDH levels, reflecting increased LDHA activity, correlate with poor prognosis across multiple solid tumors and predict response to anti-angiogenic and anti-programmed cell death protein 1 (PD-1) therapies (Gu and Yang, 2023; Marmorino et al., 2017; Petrelli et al., 2015; Wang X. et al., 2019; Yu et al., 2017; Zhang Y. et al., 2022; Zhang et al., 2023). Beyond energy production, tumor-intrinsic glycolytic programs activate inflammatory and metastatic circuits. For example, the 6-phosphofructo-2-kinase/fructose-2,6-bisphosphatase 3 (PFKFB3)–NF-κB axis induces secretion of neutrophil-attracting chemokines (CXCL2 and CXCL8) and pro-metastatic mediators such as oncostatin M (Peng et al., 2020; Que et al., 2022; Shaul and Fridlender, 2019; Wang L. et al., 2023). In colorectal cancer liver metastases, G protein-coupled receptor 37 (GPR37)-driven glycolysis and LDHA upregulation promote histone lactylation (H3K18la), epigenetically enhancing CXCL1 and CXCL5 expression and establishing a feed-forward loop that amplifies neutrophil recruitment and metastatic dissemination (Zhou et al., 2023).

The excessive production of lactate and protons acidifies the TME and acts as a potent signaling mechanism. Lactate signals through receptors such as GPR81 and reinforces immunosuppressive circuits by promoting PD-L1 expression on myeloid cells, M2 macrophage polarization, myeloid-derived suppressor cell (MDSC) differentiation, regulatory T cell (Treg) expansion, and dendritic cell (DC) dysfunction (Claps et al., 2022; Mishra and Banerjee, 2019; Kao et al., 2022; Zhang H. et al., 2024; Wang Z. H. et al., 2021). Hypoxia, driven by abnormal vascularization and high oxygen consumption, further stabilizes HIF-1α, reinforcing glycolytic dominance and sustaining the acidic milieu (Miao et al., 2024; Roy S. et al., 2020). In addition to glucose addiction, tumors remodel mitochondrial metabolism and tricarboxylic acid (TCA) cycle flux to sustain biosynthesis, redox balance, and immune evasion. Contrary to the original view of mitochondrial dysfunction as a passive consequence of the Warburg effect, tumor cells retain functional and highly adaptable mitochondria that support both biosynthetic demands and immune escape mechanisms (Klein et al., 2020). In many cancers, mitochondrial oxidative phosphorylation (OXPHOS) remains active or is selectively upregulated, allowing tumor cells to sustain ATP production, redox balance, and metabolic flexibility under fluctuating nutrient and oxygen conditions, thereby conferring a survival advantage within the TME (Klein et al., 2020). Mitochondrial metabolism directly contributes to immune evasion through multiple interconnected mechanisms. Elevated mitochondrial respiration increases oxygen consumption, exacerbating local hypoxia and reinforcing HIF-1α-dependent transcriptional programs that promote immunosuppressive signaling and tumor survival. In parallel, mitochondrial reactive oxygen species (ROS), produced at elevated levels in cancer cells, function as signaling mediators that activate oncogenic and stress-response pathways, further sustaining tumor progression while indirectly impairing antitumor immune responses (Klein et al., 2020). Moreover, mitochondrial DNA mutations and electron transport chain alterations can amplify ROS production in a feed-forward loop, thereby enhancing tumor adaptation and resistance to immune-mediated clearance. Importantly, mitochondrial rewiring also intersects with metabolic competition and immune checkpoint regulation. Increased tumor oxidative metabolism has been associated with reduced antitumor immunity and resistance to immune checkpoint blockade, partly through the creation of hypoxic and nutrient-depleted niches that limit immune cell function. In addition, mitochondrial-driven metabolic programs can modulate the expression of immune checkpoint molecules and influence the balance between tumor-promoting and tumor-suppressive signals within the TME, further consolidating immune escape (Klein et al., 2020).

**FIGURE 1:**
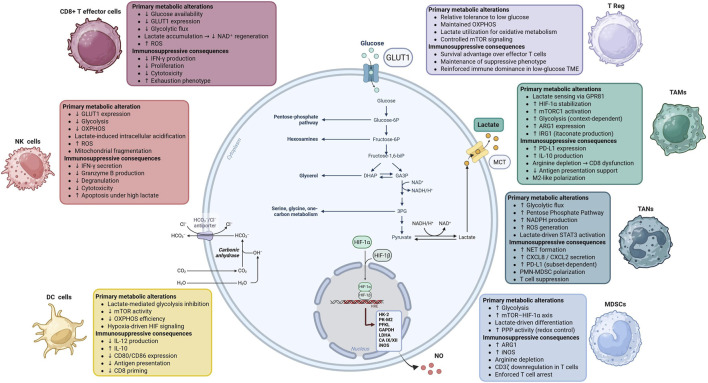
Metabolic war zone: glucose scarcity, lactate flooding, and hypoxic rewiring in tumor–immune crosstalk. Schematic overview of how tumor-driven glucose consumption, lactate accumulation, and hypoxia reshape metabolic competition within the tumor microenvironment, impairing effector immune-cell function while reinforcing immunosuppressive and tumor-adaptive programs. Created in BioRender. Cannito S. (2026) (https://BioRender.com/bakc29f).

Oncogenic KRAS signaling enhances citrate utilization and promotes the secretion of cytokines such as granulocyte–macrophage colony-stimulating factor (GM-CSF), which reprogram myeloid cell metabolism through PI3K/AKT activation and increased arginase-1 (Arg1) expression (Boyer et al., 2022). Tumor cells also compete with immune populations for glutamine and other nutrients, impairing antigen presentation and effector differentiation through mechanisms such as folliculin (FLCN)–transcription factor EB (TFEB) axis disruption (Guo et al., 2023). To avoid redundancy, the major metabolic pressures generated by this tumor program—namely, glucose deprivation, lactate accumulation, hypoxia, HIF-1α stabilization, and mTOR-centered nutrient sensing—should be considered shared regulatory axes across the immune populations discussed below. The following sections, therefore, emphasize cell-specific adaptations and consequences rather than restating the same core mechanisms for each immune subset. Collectively, tumor metabolic rewiring establishes a glucose-deprived, lactate-rich, hypoxic, and nutrient-competitive niche that selectively disadvantages effector immunity while favoring metabolically adaptable suppressive populations, thereby reinforcing immune evasion and tumor progression (Chapman and Chi, 2024; Kao et al., 2022; Zhang H. et al., 2024). Figure 1 provides an overview of the main metabolic and immunologic interactions discussed in this chapter.

### 2.1 Starved on the frontline: metabolic and mitochondrial fragility of NK cells in a glucose-depleted TME

Natural killer cell (NK) anti-tumor activity is highly dependent on glucose metabolism, coordinated glycolysis, and oxidative phosphorylation (OXPHOS) (Borde and Matosevic, 2023; Yang et al., 2023). Within glucose-depleted TMEs, defined by the shared metabolic constraints outlined above, intratumoral glucose levels can fall dramatically (to ∼20 mg/L), leading to reduced cytotoxic capacity (Hirayama et al., 2009). NK cells isolated from ovarian cancer ascites exhibit reduced GLUT1 expression, diminished glycolysis and oxidative respiration, lower ATP production, decreased nutrient transporter expression, and impaired degranulation (Poznanski et al., 2021). Mechanistically, tumor-associated upregulation of fructose-1,6-bisphosphatase 1 (FBP1) suppresses NK glycolysis. Pharmacologic inhibition of FBP1 (MB05032) restores glycolytic rates and improves tumor-infiltrating NK cell viability in murine lung carcinoma models, although late-stage dysfunction may become irreversible (Han et al., 2021; Cong et al., 2018). Sterol regulatory element-binding protein (SREBP) functions as a central regulator of NK glycolysis and OXPHOS, sustaining cytotoxicity, IFN-γ, and granzyme B (GZMB) production. Tumor-enriched metabolites such as 27-hydroxycholesterol disrupt SREBP activity and dampen NK metabolic competence (Abdalkareem Jasim et al., 2022; Assmann et al., 2017; Huang et al., 2020; O’Brien and Finlay, 2019; Rossin et al., 2019).

Within this same hostile metabolic context, hypoxia induces mitochondrial fragmentation, ROS accumulation, and downregulation of activating receptors, including CD16 and NK group 2 member D (NKG2D), partly linked to altered NO–MAPK1 signaling (Parodi et al., 2018; Teng et al., 2020; Wu et al., 2022; Zheng et al., 2019). HIF-1α plays context-dependent roles: under hypoxia, stable HIF-1α promotes glycolytic adaptation and may protect NK cells from apoptosis (Cluff et al., 2022; Lim et al., 2021a; Pelletier et al., 2023), whereas genetic deletion enhances NK activation and IFN-γ production in certain models (Ali et al., 2015; Krzywinska et al., 2017; Ni et al., 2020).

Likewise, lactate-driven stress further aggravates NK dysfunction. Tumor-derived lactic acid reduces IFN-γ secretion and cytotoxicity, lowers intracellular pH, increases mitochondrial stress and reactive oxygen species (ROS), and induces apoptosis at concentrations >20 mM (Brand et al., 2016; Harmon et al., 2019; Terrén et al., 2019). Lactate-mediated acidification inhibits nuclear factor of activated T-cell (NFAT) transcriptional activity, further suppressing IFN-γ expression (Brand et al., 2016). In pancreatic cancer, the SIX homeobox 1 (SIX1)/LDHA axis enhances lactate accumulation and NK inhibition (Ge et al., 2023).

In addition to these metabolic constraints, mitochondrial rewiring represents a critical determinant of NK cell dysfunction within the TME. NK cell cytotoxicity depends on the integration of glycolysis and OXPHOS, with mitochondria regulating ATP production and redox balance. Tumor-driven metabolic stress, including hypoxia, nutrient deprivation, and lactate accumulation, disrupts mitochondrial integrity, leading to reduced respiratory capacity and excessive production of reactive oxygen species (ROS). While physiological ROS contribute to immune signaling, their sustained accumulation promotes oxidative stress, impairs cytotoxic function, and reduces cytokine production (Klein et al., 2020). Moreover, tumor-driven oxygen consumption and metabolic competition further exacerbate mitochondrial dysfunction, limiting NK cell persistence and effector responses. Collectively, mitochondrial alterations act as a central mechanism underlying NK cell metabolic fragility and functional exhaustion within the TME, highlighting mitochondrial metabolism as a potential target for restoring NK cell-mediated antitumor immunity.

Cytokine modulation can partially restore metabolic competence. IL-21 enhances STAT3 signaling, maximal glycolytic capacity, CD98/CD71 expression, granularity, and cytotoxicity, demonstrating clinical benefit in metastatic melanoma and renal cell carcinoma (Davis et al., 2015; Petrella et al., 2012; Thompson et al., 2008). Thus, tumor metabolic rewiring profoundly compromises NK metabolic fitness through glucose deprivation, hypoxia, oxidative stress, and lactic acidosis, ultimately driving mitochondrial dysfunction that limits bioenergetic capacity, disrupts redox homeostasis, and impairs NK cell effector responses.

### 2.2 Fueling the fire: glycolytic amplification and mitochondrial adaptation in TAN

Glucose metabolism is a central driver of tumor-associated neutrophil (TAN) metabolic rewiring within the TME (Huang et al., 2025). Within the shared conditions of hypoxia, lactate accumulation, and nutrient competition established above, TANs adopt a rewired glycolytic program that supports survival and pro-tumor functions (Huang et al., 2025; Lin et al., 2025). Glycolysis-associated inflammatory pathways, such as PFKFB3–NF-κB, induce secretion of CXCL chemokines and pro-metastatic mediators (Peng et al., 2020; Que et al., 2022; Shaul and Fridlender, 2019; Wang L. et al., 2023).

In hepatocellular carcinoma (HCC), TANs exhibit enhanced glycolysis and pentose phosphate pathway activity, promoting ROS-dependent Neutrophil Extracellular Traps (NET) formation (Jiang et al., 2022). NET formation depends on glycolytic flux and NADPH production via glucose-6-phosphate dehydrogenase (G6PD) rather than mitochondrial oxidation (Azevedo et al., 2015; Rodríguez-Espinosa et al., 2015). Glycolytic rewiring enhances degranulation, cytokine secretion, vascular permeability, and tissue remodeling (Chen J. et al., 2023; Rodriguez-Rosales et al., 2021).

Clinically, increased GLUT1 expression promotes tumor growth and radiotherapy resistance in lung adenocarcinoma, while GLUT1 deletion delays progression (Ancey et al., 2021; Huang et al., 2025). Glycolysis-high TAN subsets identified by single-cell analysis correlate with immunosuppressive phenotypes and poor prognosis (Huang et al., 2025; Wang L. et al., 2023; Chen S. et al., 2023; Wang Y. et al., 2023c).

Beyond glycolysis, TANs engage mitochondrial metabolism. Aconitate decarboxylase 1 (Acod1) induction converts cis-aconitate to itaconate and promotes ferroptosis resistance via GM-CSF–JAK/STAT5–C/EBPβ–Nrf2 signaling (Zhao et al., 2023; Huang et al., 2025; Lin et al., 2025). DLST upregulation supports OXPHOS and suppressive marker expression (Lin et al., 2025). Glutamine- and fatty acid-fueled mitochondrial ROS further sustain NET formation and immune evasion (Rogers and DeBerardinis, 2021). Lactate-driven activation through the GPR81–mTOR–HIF-1α–STAT3 axis promotes polymorphonuclear (PMN)-MDSC polarization (Huang et al., 2025).

In addition, mitochondrial rewiring provides an additional regulatory layer shaping TAN function within the TME. Although TANs are predominantly glycolytic, tumor-driven metabolic stress modulates mitochondrial activity, contributing to redox signaling and cellular adaptation. Increased mitochondrial ROS production acts not only as a byproduct of metabolic stress but also as a signaling mediator that reinforces pro-tumoral functions, including survival, inflammatory activation, and immune suppression (Klein et al., 2020). Furthermore, hypoxia-driven pathways intersect with mitochondrial metabolism to sustain TAN persistence within oxygen- and nutrient-limited niches, supporting their functional plasticity and tumor-promoting activity (Klein et al., 2020; Tsai C. H. et al., 2023).

Thus, TANs integrate glycolytic amplification and TCA-linked mitochondrial adaptations to reinforce tumor progression and immune suppression.

### 2.3 Between inflammation and suppression: dynamic glycolysis–TCA rewiring in tumor-associated macrophages

Tumor-associated macrophages (TAMs) undergo extensive metabolic reprogramming within the TME, where nutrient scarcity, hypoxia, acidosis, and tumor-derived soluble factors dynamically shape their phenotype and function (Wang A. et al., 2019). Although macrophage metabolism has historically been framed within the M1–M2 paradigm, TAMs *in vivo* exhibit substantial metabolic heterogeneity that reflects integration of inflammatory signals, tumor-derived metabolites, and nutrient competition.

Classical inflammatory activation through TLR signaling (particularly TLR4 engagement by LPS) induces a rapid increase in aerobic glycolysis via the PI3K/AKT cascade, accompanied by enhanced GLUT1 expression, increased lactate production, and upregulation of activation markers (Freemerman et al., 2014; Krawczyk et al., 2010). This glycolytic shift is tightly linked to M1-like polarization and depends on TLR/NF-κB and PI3K–AKT pathways, as well as metabolic regulators including PFKFB3 (Hamidzadeh et al., 2017; Wang S. et al., 2019; Zhong et al., 2023). Inhibition of PFKFB3 suppresses IFN-γ-induced glycolysis and limits M1 polarization, underscoring the functional importance of sustained glycolytic flux in inflammatory macrophage activation (Wang S. et al., 2019).

Concomitant with enhanced glycolysis, inflammatory macrophages exhibit remodeling of the TCA cycle. A metabolic “break” at isocitrate dehydrogenase (IDH) diverts citrate from canonical OXPHOS toward itaconate production, a process described as TCA cycle rewiring (Jha et al., 2015; Seim et al., 2019). Accumulated succinate stabilizes HIF-1α and promotes IL-1β production (Tannahill et al., 2013; Selak et al., 2005), while citrate export from mitochondria fuels NADPH generation and histone acetylation required for inflammatory gene expression (Balsa et al., 2020; Billingham et al., 2022). Importantly, early inflammatory activation requires intact TCA cycle activity and OXPHOS, whereas sustained stimulation progressively disrupts mitochondrial respiration and increases dependence on aerobic glycolysis (Seim et al., 2019).

Within tumors, however, TAM metabolism does not strictly follow the classical M1 glycolysis versus M2 OXPHOS dichotomy. In several tumor contexts, TAMs display high glycolytic activity, in some cases exceeding that of tumor cells, largely driven by mTORC1 signaling and intense nutrient competition within the TME (Reinfeld et al., 2021). Tumor acidosis further stimulates glycolysis while suppressing oxidative metabolism, thereby promoting an immunosuppressive M2-like phenotype (He et al., 2021). Tumor-derived exosomes carrying PKM2 enhance glycolytic flux in TAMs, increasing acetyl-CoA production, histone acetylation, and STAT3 phosphorylation, thereby reinforcing M2-like differentiation and establishing protumoral feedback loops (Brioschi et al., 2023).

Lactate accumulation in the TME promotes M2-like polarization through GPR81 signaling and enhances PD-L1 expression on TAMs. Consistently, inhibition of glycolysis with 2-deoxy-D-glucose (2-DG) impairs M2-like TAM polarization under glucose-restricted conditions, indicating that immunosuppressive TAMs can remain highly dependent on glycolytic metabolism *in vivo* (Zhao et al., 2017).

Tumor-derived metabolites further reshape mitochondrial metabolism in TAMs. Branched-chain ketoacids modulate macrophage polarization by altering TCA intermediates and polyamine metabolism. Oncogenic KRAS-driven tumors secrete GM-CSF, activating PI3K/AKT signaling in TAMs and enhancing citrate breakdown and Arg1 expression, leading to the accumulation of immunosuppressive metabolites such as ornithine (Boyer et al., 2022). Induction of Acod1 and subsequent itaconate production further influence OXPHOS, ROS generation, and inflammatory gene expression programs (Chen Y. J. et al., 2023; O’Neill and Artyomov, 2019).

In addition to intrinsic metabolic rewiring, emerging evidence indicates that mitochondrial dynamics and intercellular mitochondrial transfer represent critical regulators of TAM polarization within the TME. Tumor–stromal and tumor–immune interactions can facilitate the transfer of functional mitochondria or mitochondrial components, leading to metabolic reprogramming of recipient macrophages and promoting immunosuppressive phenotypes (Liu et al., 2025). In particular, mitochondrial acquisition has been associated with enhanced oxidative metabolism, reduced inflammatory signaling, and impaired antigen presentation, collectively favoring a shift away from pro-inflammatory M1 states toward M2-like polarization.

Mechanistically, mitochondrial rewiring alters redox balance and metabolic signaling pathways that govern macrophage fate. Changes in mitochondrial function and reactive oxygen species (ROS) production can suppress pro-inflammatory programs while reinforcing pathways associated with tissue remodeling, immune tolerance, and tumor progression. Furthermore, mitochondrial transfer has been shown to modulate immune cell metabolism and function within the TME, contributing to immune evasion by dampening cytotoxic immune responses and promoting suppressive myeloid phenotypes (Liu et al., 2025; Zhao H. et al, 2022).

Single-cell transcriptomic analyses reveal metabolically distinct TAM subsets with differential reliance on glycolysis, oxidative metabolism, and arginine pathways, highlighting spatial and functional heterogeneity across tumor types (Geeraerts et al., 2021; Müller et al., 2017). Thus, TAMs integrate glycolytic flux, TCA cycle remodeling, and mitochondrial plasticity to dynamically respond to tumor-derived metabolic cues.

### 2.4 Silencing the sentinels: lactate, hypoxia, and glycolytic suppression of dendritic cell immunogenicity

Within the TME, dendritic cell recruitment and activation are shaped by immune crosstalk yet constrained by tumor-driven metabolic pressures. NK cells sustain the accumulation and expansion of conventional type 1 DCs (cDC1s) through FMS-like tyrosine kinase 3 ligand (FLT3L) and chemokines XCL1 and CCL5, while tumor-derived CCL4 promotes cDC1 recruitment, an axis associated with improved responses to anti-PD-1 therapy in melanoma (Barry et al., 2018; Böttcher et al., 2018).

Following antigen uptake via receptors such as CLEC9A, which recognizes exposed F-actin, cDC1s undergo maturation characterized by upregulation of MHC-I/II, CD80, CD86, CD40, and CCR7, along with IL-12 and type I/III interferon production. These changes enable migration to tumor-draining lymph nodes and cross-priming of naïve CD8^+^ T cells via TLR3–TRIF and cGAS–STING pathways (Giampazolias et al., 2021; Jelinek et al., 2011; Sancho et al., 2009; Woo et al., 2014; Xu et al., 2017).

However, tumor-derived lactic acid suppresses IL-12 production and DC-dependent antigen presentation via GPR81 signaling. Lactate promotes IL-10 and indoleamine 2,3-dioxygenase 1 (IDO1) expression, inhibits glycolysis in plasmacytoid DCs, and favors Treg differentiation (Bajwa et al., 2016; Brown et al., 2020; Feichtinger and Lang, 2019; Gottfried et al., 2006; Ranganathan et al., 2018; Raychaudhuri et al., 2019; Zhao et al., 2018).

Hypoxia-induced HIF-1α further shapes DC function by promoting tolerogenic differentiation and impairing Th1 polarization. Adenosine accumulation in the TME acts through the A2B receptor to suppress IL-12 production and induce expression of IL-10, COX-2, IDO1, ARG1/2, and VEGF, collectively reducing DC immunostimulatory capacity and impairing cytotoxic CD8^+^ T-cell activation (Addi et al., 2008; Challier et al., 2013; Chaudhari et al., 2015; Flück et al., 2016; Haskó et al., 2009; Kheshtchin et al., 2016; Novitskiy et al., 2008; Omar et al., 2009; Panther et al., 2001; Perrot et al., 2019; Silva-Vilches et al., 2018; Wilson et al., 2009; Zarek et al., 2008).

Thus, even in the absence of lipid metabolic discussion, tumor-driven glycolytic stress, lactate signaling, hypoxia, and adenosine collectively convert DCs from immunogenic antigen-presenting cells into tolerogenic regulators.

### 2.5 Running on empty: glycolytic commitment and metabolic exhaustion of effector T cells in the TME

Naïve and memory CD8^+^ T cells primarily rely on OXPHOS to sustain basal homeostasis. Upon antigen stimulation, however, activated CD8^+^ and CD4^+^ effector T cells undergo a profound metabolic shift toward aerobic glycolysis, adopting a metabolic profile that closely resembles that of proliferating cancer cells (Hu Z. et al., 2019; Liao et al., 2024; Menk et al., 2018; Pearce et al., 2013; Wang R. et al., 2025; Zhang H. et al., 2024). This glycolytic commitment supports rapid biomass accumulation, clonal expansion, and effector molecule production. High glycolytic activity promotes CD8^+^ effector differentiation and IFN-γ production (Cao et al., 2023), along with secretion of IL-2 and IL-17 in CD4^+^ subsets (Lv et al., 2025; Pecchillo Cimmino et al., 2024; Procaccini et al., 2021).

T cell receptor (TCR) engagement and co-stimulatory signaling activate NF-κB and NFAT pathways, inducing transcription factors MYC and HIF-1α, which coordinate the upregulation of glycolytic genes, including GLUT1, HK2, LDHA, and PKM (Finlay et al., 2012; Gnanaprakasam et al., 2017; Johnson et al., 2018; Wang et al., 2016; Huang H., et al 2021). mTORC1 functions as a central metabolic integrator during antigen-driven activation, coupling nutrient sensing to glycolytic reprogramming and anabolic metabolism (Böttcher et al., 2016; Chen Y. et al., 2023; Cheng et al., 2022; Yang et al., 2013; Zeng et al., 2016). Sustained mTOR activity ensures the maintenance of glycolytic flux, whereas reduced mTOR signaling suppresses glycolysis and limits effector differentiation (Song et al., 2018).

Beyond ATP production, glycolysis feeds multiple metabolic branches that shape effector function. The pentose phosphate pathway (PPP) generates NADPH required for ROS detoxification and redox homeostasis (Bender et al., 2023; Huang et al., 2022; Silic-Benussi et al., 2022). PPP flux increases during CD4^+^ T-cell activation and supports biosynthetic demands. The hexosamine biosynthesis pathway (HBP) generates UDP-N-acetylglucosamine (GlcNAc), which sustains O-GlcNAcylation of key transcriptional and signaling proteins required for effector differentiation (Lam et al., 2021; Swamy et al., 2016). Furthermore, TCA cycle intermediates such as α-ketoglutarate (α-KG) and succinate act as signaling metabolites that regulate CD4^+^ T-cell lineage decisions and effector responses (Gudgeon et al., 2022; Matias et al., 2021). Elevated acetyl-CoA levels can also support histone acetylation and IFN-γ production under nutrient-limited conditions (Pecchillo Cimmino et al., 2024).

Within the nutrient competition established in Section 2, enhanced tumor glycolysis creates a state of nutrient competition in which tumor cells outcompete T cells for glucose (Ho et al., 2015; Liu et al., 2021b; Leone et al., 2019). Glucose deprivation reduces intracellular phosphoenolpyruvate (PEP) levels. PEP is not merely a glycolytic intermediate but a regulator of Ca^2+^–NFAT signaling: reduced PEP impairs calcium flux, diminishes NFAT activation, and consequently decreases IFN-γ production (Ho et al., 2015). In parallel, low glucose availability activates AMPK and inhibits mTORC1 signaling, further suppressing glycolytic flux and effector differentiation (He et al., 2021; Song et al., 2018).

Lactate-driven metabolic stress adds a second major layer of suppression. Elevated extracellular lactate reduces the intracellular NAD^+^/NADH ratio in T cells, blocking glycolysis at the level of glyceraldehyde-3-phosphate dehydrogenase (GAPDH) and thereby limiting ATP production and biosynthetic capacity (Sun et al., 2023;Kao et al., 2022; Zhang H. et al., 2024). Acidification of the TME further exacerbates metabolic stress, impairing cytokine production and cytotoxic activity.

Under these chronic metabolic constraints, tumor-infiltrating lymphocytes (TILs) shift toward increased reliance on OXPHOS. However, sustained mitochondrial engagement under conditions of limited substrate availability results in mitochondrial depolarization, accumulation of dysfunctional mitochondria, and increased ROS production (Chapman and Chi, 2024). This mitochondrial stress contributes to T-cell dysfunction and exhaustion, characterized by reduced cytokine production, impaired cytotoxicity, and diminished proliferative capacity.

Emerging evidence indicates that mitochondrial dysfunction in T cells is not solely a consequence of metabolic stress but can be actively imposed by tumor cells through intercellular mitochondrial transfer. Tumor cells can deliver dysfunctional mitochondria carrying pathogenic mitochondrial DNA (mtDNA) mutations into TILs via tunneling nanotubes and extracellular vesicles, directly impairing mitochondrial integrity and oxidative phosphorylation (OXPHOS) capacity (Ikeda et al., 2025). This process disrupts ATP production and metabolic fitness, promoting a state of bioenergetic insufficiency that culminates in terminal T-cell exhaustion and impaired antitumor immunity.

Mechanistically, tumor-derived mitochondria can evade mitophagy and progressively replace endogenous mitochondrial networks within T cells, leading to sustained loss of mitochondrial membrane potential, accumulation of oxidative stress, and defective metabolic signaling. This mitochondrial rewiring is associated with reduced cytokine production, impaired memory formation, and decreased proliferative capacity, all hallmarks of exhausted T cells. Importantly, this process has been linked to resistance to immune checkpoint blockade, highlighting mitochondrial transfer as a direct mechanism of immune evasion within the TME (Ikeda et al., 2025).

Metabolic rescue strategies highlight the mechanistic centrality of glycolytic intermediates. Overexpression of phosphoenolpyruvate carboxykinase 1 restores PEP levels and enhances tumor suppression in melanoma models (Ho et al., 2015; Lv et al., 2025), demonstrating that metabolic rewiring can directly modulate effector function.

Effector T-cell metabolism is therefore governed by tightly coordinated glycolytic commitment, branching biosynthetic pathways, and mitochondrial integration. Tumor-induced glucose deprivation and lactate accumulation disrupt these networks at multiple regulatory nodes (including mTOR signaling, PEP–NFAT coupling, NAD^+^ regeneration, and mitochondrial integrity), thereby driving progressive effector dysfunction and exhaustion within the TME.

### 2.6 A not-so-sweet balance: glycolysis and OXPHOS adaptation of Tregs in the TME

Tregs display a distinctive metabolic configuration characterized by tightly regulated glycolytic flux and a predominant reliance on mitochondrial OXPHOS. While glycolysis is required to sustain Treg proliferation, particularly downstream of mTOR signaling, excessive glycolytic activity impairs their suppressive capacity, indicating that glycolytic flux must be finely balanced rather than maximized (Charbonnier et al., 2019; Mossmann et al., 2018; Rao et al., 2021). Increased expression of GLUT1 enhances proliferative expansion, yet sustained high glycolytic rates destabilize suppressive programming, demonstrating a functional segregation between the metabolic pathways controlling growth and those maintaining immune regulation.

Notably, Tregs exhibit reduced basal mTOR activity compared with conventional effector T cells, partly due to activation of protein phosphatase 2A (PP2A) and Foxp3-mediated modulation of intracellular signaling (Apostolidis et al., 2016). This restrained mTOR signaling limits excessive glycolytic commitment and preserves suppressive stability. Foxp3 directly inhibits MYC signaling and attenuates AKT–mTOR pathway activation, thereby suppressing glycolytic gene expression and promoting mitochondrial respiration (Chapman et al., 2020; Wang et al., 2018; Angelin et al., 2017; Chou et al., 2021; Sarkar et al., 2021).

Functionally, Tregs rely predominantly on mitochondrial metabolism. Experimental inhibition of mitochondrial complex III preserves Treg numbers but significantly impairs suppressive capacity, demonstrating that OXPHOS is not merely supportive but mechanistically required for regulatory function (Weinberg et al., 2019). This metabolic compartmentalization establishes glycolysis as primarily supporting proliferation, whereas mitochondrial respiration sustains suppressive activity. Within the TME, this metabolic architecture confers a selective advantage.

Unlike effector T cells, Tregs tolerate elevated lactate concentrations and low glucose availability. They maintain NAD^+^ regeneration by coupling the electron transport chain to the TCA cycle and can utilize lactate-derived carbon to sustain oxidative metabolism under hypoxic and nutrient-restricted conditions (Angelin et al., 2017). Pharmacologic reprogramming toward glycolysis, such as metformin-induced metabolic shifts, weakens Tregs’ suppressive function, further underscoring the functional dominance of OXPHOS in regulatory stability (Jung et al., 2020). Accordingly, mitochondrial metabolism plays a central role in stabilizing Treg suppressive activity within the tumor microenvironment. Tregs preferentially rely on OXPHOS and mitochondrial redox balance to sustain long-term function under metabolic stress, in contrast to effector T cells, which are more sensitive to mitochondrial dysfunction. Mitochondrial signaling, including controlled reactive oxygen species (ROS) production, supports immune tolerance and reinforces suppressive programs (Klein et al., 2020). Furthermore, emerging evidence suggests that tumor-driven mitochondrial dynamics, including intercellular mitochondrial transfer, may contribute to metabolic adaptation of immune cells by reshaping mitochondrial function and bioenergetic capacity, thereby promoting immunosuppressive phenotypes and sustaining Treg-mediated immune evasion within the TME (Liu et al., 2025).

### 2.7 When glucose dictates suppression: metabolic control of MDSC plasticity

Glucose metabolism represents a central determinant of MDSC differentiation, expansion, and suppressive function within the TME. In the hypoxic TME, stabilization of HIF-1α promotes the differentiation of bone marrow-derived progenitors into MDSCs and enhances their recruitment and retention in tumors (Kumar et al., 2016; Lu Y. et al., 2018). HIF-1α not only supports survival under low oxygen tension but also directly coordinates transcriptional programs that reinforce glycolytic commitment and immunosuppressive mediator expression.

Activated MDSCs exhibit enhanced glycolysis and increased PPP activity, acquiring a Warburg-like metabolic phenotype (Veglia et al., 2021). This metabolic reprogramming is primarily regulated by the PI3K–AKT–mTOR axis (Gabrilovich, 2017; Hu et al., 2020; Wang Y. et al., 2020). mTOR activation sustains glycolytic flux through downstream effectors, including HIF-1α, c-Myc, and p53, which transcriptionally upregulate GLUT family members and glycolytic enzymes such as LDH, thereby reinforcing lactate production and maintaining NAD^+^ regeneration (Gabrilovich, 2017; Li et al., 2017; Hu et al., 2020; Wang Y. et al., 2020).

Pharmacologic inhibition of mTOR signaling reduces MDSC accumulation within tumors and restores anti-tumor T-cell responses, underscoring the centrality of mTOR-driven glycolysis in maintaining suppressive capacity (Deng et al., 2018; Fultang et al., 2019b; Li et al., 2021).

Glycolytic intermediates directly contribute to MDSC survival and immunoregulatory activity. PEP limits oxidative stress and prevents apoptosis, linking glycolytic flux to redox homeostasis (Gabrilovich, 2017; Jian et al., 2017; Okła, 2023). Concurrently, enhanced PPP activity generates NADPH, which supports ROS buffering and sustains suppressive mediator production. Thus, glycolysis in MDSCs is not merely bioenergetic but structurally integrated with antioxidant defense and immunosuppressive signaling.

As in the broader glycolytic framework above, lactate accumulation within the TME further reinforces MDSC function. Tumor-derived lactate enhances MDSC differentiation and suppressive programming (Payen et al., 2020; Wang Y. et al., 2020).

Regulation of lactate transporters modulates MDSC metabolic adaptation and functional differentiation, indicating that lactate uptake and efflux mechanisms contribute to the shaping of suppressive phenotypes (Hayes et al., 2021; Zhao J. L. et al., 2022). Through these mechanisms, lactate operates both as a metabolic substrate and as a signaling metabolite that stabilizes immunosuppressive circuits.

Importantly, although glycolysis dominates early activation and expansion phases, MDSCs display metabolic flexibility during tumor progression. In advanced tumors, MDSCs can shift toward increased reliance on OXPHOS and fatty acid oxidation (FAO), further enhancing their suppressive potency (Mohammadpour et al., 2021; Lian et al., 2022). This transition reflects adaptive plasticity rather than a simple metabolic switch, allowing MDSCs to maintain function under fluctuating nutrient and oxygen conditions. In this context, mitochondrial metabolism provides a critical regulatory layer sustaining MDSC suppressive activity. Enhanced OXPHOS supports ATP production and redox balance, while mitochondrial-derived reactive oxygen species (ROS) act as signaling mediators, reinforcing immunosuppressive programs and T-cell inhibition (Klein et al., 2020). Moreover, emerging evidence suggests that tumor-driven mitochondrial dynamics, including intercellular mitochondrial transfer, can further promote metabolic adaptation and stability of suppressive myeloid phenotypes within the TME, contributing to immune evasion (Liu et al., 2025; Ikeda et al., 2025).

Collectively, MDSC metabolic programming is orchestrated by the PI3K–AKT–mTOR–HIF-1α axis and integrates glycolysis, PPP-mediated redox control, lactate signaling, and context-dependent engagement of mitochondrial metabolism. This coordinated rewiring establishes MDSCs as metabolically adaptable suppressor cells that efficiently sustain immune evasion in progressively hostile tumor environments (Zhang H. et al., 2024; Wang R. et al., 2025).

## 3 A greasy business: lipogenesis, cholesterol flux, and oxidative lipid stress in the TM

Beyond glycolysis, tumor cells profoundly reconfigure lipid metabolism to sustain growth, structural remodeling, and immune evasion. The TME is frequently enriched in fatty acids (FAs), cholesterol, lipoproteins, oxysterols, and complex sphingolipids as a consequence of robust *de novo* lipogenesis within tumor cells and the metabolic contribution of adipocytes and stromal cells (De Martino et al., 2024; Kao et al., 2022; Zhang H. et al., 2024).

At the intracellular level, tumor cells activate SREBP1/2, which transcriptionally upregulates enzymes such as FA synthase (FASN) and acetyl-CoA carboxylase (ACC1), thereby driving FA and cholesterol synthesis to sustain membrane biogenesis, rapid proliferation, and redox homeostasis (Ma and Zhang, 2021; Meng et al., 2024; Yan C. et al., 2023; Yang et al., 2016). FASN-driven lipid synthesis supports phospholipid and cholesterol production required for membrane expansion and oncogenic signaling platforms.

Tumor cells also remodel the extracellular lipid environment, releasing bioactive lipid species that can act on neighboring immune and stromal cells within the TME. These signals contribute to the establishment of pro-tumoral and immunosuppressive circuits by altering myeloid-cell recruitment, activation, and metabolic polarization (Baek et al., 2017; Coffelt et al., 2015; Raccosta et al., 2013; Goossens et al., 2019; Puglisi et al., 2015; Steggerda et al., 2017; Di Conza et al., 2021).

**FIGURE 2:**
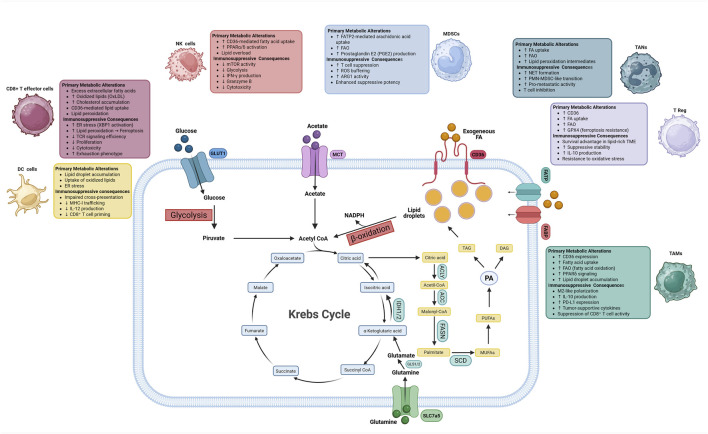
Fat chance: lipid rewiring and immune subversion in tumor–immune crosstalk. Schematic representation of how altered lipid synthesis, uptake, storage, and cholesterol handling in tumor and immune cells promote immune dysfunction, suppress antigen presentation, and support tumor progression within the tumor microenvironment. Created in BioRender. Cannito S. (2026) (https://BioRender.com/bakc29f).

Through coordinated lipid synthesis, uptake, efflux, and oxidation, tumors create a lipid-rich and oxidatively stressed microenvironment that imposes selective metabolic pressure on infiltrating immune cells, favoring populations capable of adapting to lipid overload and ferroptosis-prone conditions (De Martino et al., 2024; Kao et al., 2022). Collectively, lipid excess and its associated processes, including FAO engagement, oxysterol signaling, cholesterol remodeling, and lipid peroxidation, constitute a shared metabolic backdrop of the tumor microenvironment. The sections below, therefore, focus on the cell-specific consequences of this lipid landscape, highlighting how individual immune populations respond to a common lipid stress context. Figure 2 recapitulates the principal concepts and mechanisms described in the text.

### 3.1 Too much fuel, too little fire: lipid uptake and PPAR-driven suppression of NK cytotoxicity

NK cell function is highly sensitive to lipid availability and metabolic balance. Under conditions of systemic lipid excess, such as obesity, NK cells accumulate lipids intracellularly, resulting in impaired proliferation, reduced IFN-γ production, defective cytotoxic granule polarization, and compromised immune synapse formation (Ghaben and Scherer, 2019; Michelet et al., 2018).

Mechanistically, enhanced FA uptake via CD36 activates PPARα and PPARδ signaling, which suppresses mTOR and c-Myc activity. This suppression leads to reduced glycolysis, diminished OXPHOS, and decreased expression of perforin and IFN-γ (Ghaben and Scherer, 2019; Michelet et al., 2018). Pharmacologic inhibition of PPAR signaling restores NK cells’ metabolic competence, whereas PPAR agonists reproduce lipid-induced dysfunction.

However, lipid metabolism in NK cells is not intrinsically suppressive. Coordinated lipid catabolism contributes to ATP generation and effector molecule biosynthesis, supporting persistence in nutrient-restricted TMEs (Kobayashi et al., 2020; Poznanski et al., 2021). The SREBP axis integrates lipid synthesis with glycolytic and mitochondrial programs. SREBP activation supports IFN-γ and GZMB production through cooperation with c-Myc and polyamine biosynthesis pathways (Assmann et al., 2017; O’Brien et al., 2021). Furthermore, myocyte enhancer factor 2C (MEF2C) connects PI3K–AKT–mTORC1 signaling to SREBP-dependent lipid metabolism; MEF2C deficiency impairs cytotoxicity, a defect that is reversible through lipid supplementation (Li et al., 2024).

Thus, NK cell function depends on tightly balanced lipid metabolic programming: excessive lipid uptake suppresses effector pathways, whereas coordinated lipid biosynthesis and oxidation sustain cytotoxic fitness (Miao et al., 2024; Yang et al., 2023; Yang et al., 2025).

### 3.2 Fat-fueled and functionally twisted: FAO, oxysterol signaling, and prostaglandin-driven immunosuppression in TANs

Building upon their previously described glycolytic and mitochondrial adaptations, TANs undergo a further layer of metabolic reprogramming centered on lipid utilization. Within the lipid-rich and oxidatively stressed environment outlined above, TANs progressively increase their reliance on mitochondrial FAO to sustain OXPHOS, ROS generation, NET formation, and immunosuppressive activity (Hsu et al., 2019; Lin et al., 2025; Rice et al., 2018). In murine breast cancer models, immature c-Kit^+^ TAN subsets display enhanced lipid uptake and FAO, a metabolic configuration that promotes T-cell suppression, NETosis, and metastatic progression (Hsu et al., 2019; Rice et al., 2018).

Lipid-derived signals actively shape neutrophil phenotype. Cholesterol metabolites, including 27-hydroxycholesterol, expand neutrophil populations and enhance angiogenesis while fostering immunosuppressive microenvironments (Baek et al., 2017; Coffelt et al., 2015). Tumor-derived oxysterols recruit neutrophils into primary tumors and pre-metastatic niches via CXCR2-dependent mechanisms, thereby linking lipid signaling to chemotactic immune remodeling (Raccosta et al., 2013).

In parallel, myeloperoxidase-driven lipid peroxidation generates immunomodulatory phospholipids, establishing a functional link between lipid metabolism, redox regulation, and ferroptosis-related pathways (Veglia et al., 2017). Endogenous lipid synthesis is also required for neutrophil survival. Disruption of the FASN–peroxisomal lipid axis impairs granulopoiesis and induces ER stress-associated apoptosis (Lodhi et al., 2015). Moreover, modulation of ether lipid metabolism reduces IL-8 production, neutrophil recruitment, and NET formation, thereby restraining tumor progression and metastasis (Chen C. et al., 2024).

Through coordinated lipid uptake, oxidation, biosynthesis, and peroxidation, TANs adopt a metabolically flexible phenotype that supports tumor progression and immune suppression.

### 3.3 Fat-fed and checkpoint-friendly: lipid flux and immune checkpoint reprogramming in tumor-associated macrophages

Lipid metabolic rewiring is a central determinant of TAM heterogeneity and immune evasion. Although the classical paradigm associates pro-inflammatory macrophages with glycolysis and FA synthesis and anti-inflammatory macrophages with FAO and OXPHOS, emerging evidence indicates a far more dynamic and context-dependent metabolic architecture (Im et al., 2011).

Within the TME, TAMs increase lipid uptake via receptors, including CD36, SR-A, and SR-BI, promoting intracellular lipid accumulation and functional reprogramming (Chen Y. et al., 2022; Xiang et al., 2018). Tumor-derived long-chain FAs, frequently delivered via extracellular vesicles, upregulate CD36 expression and enhance FAO through PPARδ signaling. This metabolic shift supports IL-10 production, reinforces M2-like polarization, and promotes metastasis (Park et al., 2015; Yang P. et al., 2022). Genetic deletion or pharmacologic inhibition of CD36 restores CD8^+^ T cell activity and reduces metastatic burden (Park et al., 2015; Yang P. et al., 2022).

Lipid metabolic enzymes further coordinate TAM reprogramming. Upregulation of SREBP1 and FASN reshapes FA synthesis and is required for inflammatory signaling downstream of TLR4 and inflammasome activation (Carroll et al., 2018; Im et al., 2011). Monoacylglycerol lipase (MGLL) deficiency induces lipid accumulation and alters TAM activation states, thereby influencing CD8^+^ T cell–mediated anti-tumor responses (Xiang et al., 2018).

Alterations in FAO integrate metabolic and immune signals. Caspase-1-mediated cleavage of PPARγ suppresses medium-chain acyl-CoA dehydrogenase (MCAD) expression and FAO, promoting differentiation toward a protumoral macrophage phenotype (Galluzzi et al., 2016; Niu et al., 2017). However, genetic carnitine palmitoyltransferase 2 (CPT2) deletion or selective FAO inhibition does not fully abrogate IL-4–induced M2 gene expression, highlighting metabolic flexibility and revealing potential off-target effects of high-dose etomoxir (Divakaruni et al., 2018; Nomura et al., 2016; O’Connor et al., 2018; Raud et al., 2018).

Cholesterol dynamics further modulate TAM phenotype. Tumor-derived hyaluronic acid promotes cholesterol efflux via CD44 and ABCA1/ABCG1, activating IL-4R/STAT6/PI3K signaling and disrupting lipid rafts, thereby suppressing IFN-γR-mediated inflammatory signaling (Goossens et al., 2019; Puglisi et al., 2015; Steggerda et al., 2017). Tumor-derived glucosylceramide induces ER stress and activates IRE1/XBP1 and STAT3 signaling, thereby amplifying immunosuppressive transcriptional programs (Di Conza et al., 2021).

PGE2, generated through enhanced arachidonic acid metabolism, drives PD-L1 transcription via PKM2 and HIF1α signaling, reinforcing immune checkpoint pathways and M2-like polarization (Palsson-McDermott et al., 2017; Prima et al., 2017; Vitale et al., 2019). Single-cell analyses identify metabolically specialized TAM subsets enriched in lipid-handling programs, including FABP5^+^ and APOE^+^ lipid-associated macrophages in breast cancer and Trem2^+^ populations in metastatic niches (Wu S. Z. et al., 2021; Huggins et al., 2021; Yang Q. et al., 2021). Through coordinated lipid uptake, synthesis, oxidation, and efflux, TAMs integrate metabolic and immune checkpoint signaling pathways to sustain tumor progression (Dussold et al., 2024; Qian et al., 2024; Wang X. et al., 2025).

### 3.4 Lipid-laden and losing vigilance: ER stress and antigen presentation collapse in tumor-associated DCs

Within the TME, DCs are exposed to a lipid-rich *milieu* that profoundly alters their metabolic state and immunogenic capacity. Tumor-associated DCs accumulate excessive triglycerides, cholesterol esters, and FAs as a consequence of increased lipid uptake mediated by scavenger receptors, lipoprotein lipase, and FA-binding proteins (Arai et al., 2018; Herber et al., 2010; Sim et al., 2016; Veglia and Gabrilovich, 2017). Elevated triglyceride accumulation has been directly documented in DCs isolated from patients with advanced lung cancer, indicating that lipid overload is not merely a preclinical observation but a clinically relevant phenomenon (Arai et al., 2018).

This lipid accumulation is not metabolically inert. Instead, it induces ER stress and compromises antigen presentation capacity (Kao et al., 2022; Zhang H. et al., 2024). Mechanistically, oxidized truncated lipids accumulate within intracellular lipid bodies and bind heat shock protein 70 (HSP70), thereby trapping peptide–MHC class I complexes within late endosomal and lysosomal compartments and preventing their trafficking to the cell surface (Veglia et al., 2017). This defect directly impairs cross-presentation to CD8^+^ T cells and disrupts cytotoxic T cell priming.

The impairment of antigen presentation is further amplified by non-cell-autonomous mechanisms. Myeloperoxidase (MPO)-dependent lipid transfer from PMN-MDSCs to DCs contributes additional oxidized lipid species, reinforcing the cross-presentation blockade (Ugolini et al., 2020).

Beyond lipid storage and oxidative modification, tumor-infiltrating DCs increase FAO, a metabolic shift associated with tolerogenic programming and suppression of effector CD8^+^ T-cell activation (Zhao et al., 2018). Concurrently, activation of the mevalonate pathway and downstream small GTPases accelerates lysosomal antigen degradation, reducing the availability of intact antigen for effective MHC loading (Mullen et al., 2016; Pearce and Everts, 2015; Thwe et al., 2017). Tumor cells additionally compete with cDC1s for glutamine, impairing antigen presentation through disruption of the FLCN–TFEB axis (Guo et al., 2023).

Through coordinated lipid accumulation, ER stress induction, FAO engagement, and lysosomal reprogramming, DCs progressively transition from immunogenic antigen-presenting cells into metabolically constrained tolerogenic regulators, thereby weakening anti-tumor T cell priming.

### 3.5 Greased into exhaustion: cholesterol stress, ferroptosis, and the collapse of anti-tumor T cells

Lipid metabolism is tightly reprogrammed during T-cell activation and critically shapes effector fate within the TME. Upon activation, effector T cells upregulate SREBP1/2-driven lipid synthesis and cholesterol uptake to sustain membrane expansion, TCR clustering, and downstream signaling (Ma and Zhang, 2021; Meng et al., 2024; Yan C. et al., 2023; Yang et al., 2016). In parallel, memory T cells preferentially rely on FAO and OXPHOS for long-term persistence, a process coordinated by AMPK activation and CPT1α-mediated mitochondrial FA import (Assmann et al., 2017; Hunt et al., 2024; Lim et al., 2021b; Saibil et al., 2019; Pearce et al., 2009; Raud et al., 2018; van der Windt et al., 2012).

Within the TME, however, excessive lipid exposure becomes detrimental. Cholesterol accumulation induces ER stress through activation of X-box binding protein 1 (XBP1), which drives upregulation of immune checkpoint molecules and promotes CD8^+^ T-cell exhaustion (Ma et al., 2021; Poznanski et al., 2021). In pancreatic ductal adenocarcinoma, CH25H deficiency further elevates cholesterol levels in the TME, reduces MHC-I expression, and exacerbates T-cell dysfunction (Zheng et al., 2024).

CD36-mediated uptake of oxidized lipids constitutes a critical vulnerability. In CD8^+^ T cells, oxidized lipid uptake activates p38 signaling and induces ferroptosis, a form of iron-dependent cell death characterized by the accumulation of lipid peroxides and loss of cytotoxic function (Ma et al., 2021; Wang H. et al., 2020; Xu S. et al., 2021). Ferroptotic susceptibility links lipid overload directly to effector collapse.

Cholesterol esterification via acetyl-CoA acetyltransferase 1 (ACAT1) regulates membrane cholesterol distribution and TCR signaling strength. Inhibition of ACAT1 enhances CD8^+^ T-cell anti-tumor activity by restoring membrane cholesterol dynamics and improving TCR signaling efficiency (Pandit et al., 2023; Yang et al., 2016). Conversely, excessive cholesterol accumulation promotes cellular senescence and tumor-supportive cytokine secretion (Wang R. et al., 2023).

FA synthesis enzymes such as ACC1 and FASN further shape CD4^+^ T-cell differentiation and regulatory balance. PPARα-driven FAO enhances CD8^+^ T-cell tumor killing, particularly in combination with PD-1 blockade, whereas PD-1 signaling or leptin–STAT3-induced FAO suppresses glycolysis and limits effector responses (Lv et al., 2025; Zhang H. et al., 2024).

Thus, lipid metabolic imbalance in the TME transforms a biosynthetic requirement into a liability, driving exhaustion, ferroptosis, and functional impairment of anti-tumor T cells.

### 3.6 Feeding the silence: lipid uptake, FAO, and ferroptosis resistance in tumor-infiltrating Tregs

In contrast to effector T cells, Tregs derive a metabolic advantage from lipid-rich environments. Within tumors, Tregs upregulate SREBP activity, enhancing lipid synthesis and oxidation to sustain mitochondrial respiration, membrane remodeling, and suppressive stability (Lim et al., 2021a; Ramos et al., 2023; Yan et al., 2022). FASN activity is required for Tregs’ maturation and function; inhibition of acetyl-CoA production reduces FA accumulation and impairs proliferation (Lim et al., 2021a).

Tregs further enhance lipid uptake and FAO to maintain immunosuppressive capacity. Activation of PPARγ increases CD36 expression and CPT1-mediated FAO, strengthening suppressive responses and associated glycosylation pathways (Miao et al., 2022). Upregulation of SLC27A1 further supports lipid transport into Tregs (Miska et al., 2019). High lipid availability within the TME preferentially supports Treg persistence over effector T cells (Shan et al., 2024).

Short-chain FAs can expand Tregs by inducing IL-10 production and FoxP3 transcription via JNK1/p38 signaling pathways, although these effects remain context-dependent (Coutzac et al., 2020; Haghikia et al., 2015; Hu M. et al., 2019; Schwarz et al., 2021). Cholesterol homeostasis also influences Treg abundance, as elevated HDL levels and hypercholesterolemia correlate with increased Treg numbers (Mailer et al., 2017; Schmitz et al., 2023).

A critical distinction lies in ferroptosis resistance. Tregs upregulate glutathione peroxidase 4 (GPX4), which detoxifies lipid peroxides and protects against ferroptotic cell death, allowing survival under oxidative lipid stress that compromises effector CD8^+^ T cells (De Martino et al., 2024; Kao et al., 2022).

Through coordinated lipid synthesis, uptake, FAO engagement, and peroxide detoxification, Tregs acquire a metabolic configuration that thrives under tumor-imposed lipid stress.

### 3.7 Licensed by lipids: prostaglandin signaling and metabolic reinforcement of MDSC immunosuppression

MDSCs are profoundly shaped by lipid availability within the TME. FATP2-mediated uptake of long-chain FA, particularly arachidonic acid, fuels PGE2 synthesis in PMN-MDSCs (Veglia et al., 2019; Veglia et al., 2021). PGE2 production directly suppresses cytotoxic T-cell responses and reinforces immunosuppressive differentiation programs. Inhibition of FATP2 or blockade of PGE2 signaling reduces suppressive capacity and metastatic progression (Veglia et al., 2019; Veglia et al., 2021).

Within this same lipid-conditioned environment, lactate and lipid metabolites synergistically reinforce MDSC differentiation and metabolic adaptation (Payen et al., 2020; Wang Y. et al., 2020).

Through coordinated lipid uptake, peroxidation, and prostaglandin production, MDSCs integrate metabolic and inflammatory signals to stabilize suppressive phenotypes within lipid-rich tumor niches.

## 4 Starve and conquer: amino acid capture and immune deprivation in cancer metabolic rewiring

**FIGURE 3:**
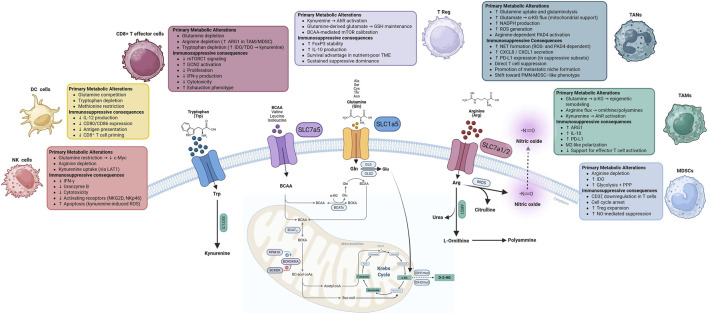
Nitrogen under siege: amino acid starvation and oncometabolite warfare in tumor–immune crosstalk. Schematic representation of how competition for amino acids and the accumulation of immunosuppressive metabolites and oncometabolites disrupt T-cell and myeloid-cell function, thereby promoting immune escape and limiting anti-tumor responses. Created in BioRender. Cannito S. (2026) (https://BioRender.com/bakc29f).

Beyond the well-characterized rewiring of glucose and lipid metabolism, tumor cells profoundly reshape amino acid metabolism to sustain proliferation, redox balance, nucleotide synthesis, epigenetic remodeling, and immune escape. Within the TME, cancer cells and tumor-associated myeloid populations actively reconfigure the availability and flux of key amino acids, creating a metabolically restrictive niche that exerts selective pressure on infiltrating lymphocytes and antigen-presenting cells (Kao et al., 2022; Wang et al., 2024; Zhang H. et al., 2024). Rather than functioning merely as substrates for protein synthesis, amino acids operate as carbon and nitrogen donors, redox regulators, epigenetic modifiers, and signaling molecules, making their depletion a powerful strategy for metabolic immune control. Altogether, amino acid competition establishes a shared nitrogen-restricted state within the TME, in which glutamine scarcity, arginine depletion, tryptophan catabolism, methionine sequestration, and variable asparagine availability act in concert to reshape immune behavior. Rather than treating these processes as isolated events in each subsection, the following paragraphs examine their cell-specific functional outcomes across distinct immune populations. The key mechanisms described in this paragraph are schematically summarized in Figure 3.

### 4.1 Glutamine: central carbon–nitrogen hub and metabolic tug-of-war

Glutamine occupies a central position in tumor metabolic rewiring. Serving both as a carbon and nitrogen donor, glutamine fuels the TCA cycle through conversion to glutamate and subsequently to α-KG, while simultaneously supporting nucleotide synthesis, lipid biosynthesis, and glutathione (GSH) production. Many tumors are, therefore, described as “glutamine-addicted,” relying heavily on glutaminolysis to sustain proliferation and maintain redox homeostasis (Wang et al., 2024; Zhang H. et al., 2024).

This addiction is reinforced by coordinated transcriptional and oncogenic regulation. Tumor cells upregulate a broad repertoire of glutamine transporters, including SLC1A5 (ASCT2), SLC7A5 (LAT1), SLC6A14, and SLC38A1/2/3/5, to maximize glutamine uptake from the extracellular *milieu*. Intracellularly, enzymes such as glutaminase (GLS), glutamate dehydrogenase (GLUD), and transaminases GOT and GPT catalyze glutamine conversion into TCA-intermediate-feeding metabolites (Wang et al., 2024; Zhang H. et al., 2024).

Oncogenic drivers, including MYC, KRAS, PI3K/mTOR, and ERBB2, further enhance glutamine uptake and catabolic flux, while loss of tumor suppressors such as PTEN, RB1, and p53 tightens cellular dependence on glutamine-derived carbon (De Martino et al., 2024; Wang et al., 2024; Zhang H. et al., 2024). As a consequence, tumor cells frequently consume glutamine at rates five to ten times higher than normal cells, generating localized depletion zones within the TME.

Glutamine deprivation markedly inhibits T-cell proliferation and reduces IL-2 and IFN-γ production. CD8^+^ memory T cells and NK cells similarly depend on glutamine for maintenance of c-Myc expression, glycolytic engagement, and cytotoxic function; glutamine scarcity reduces c-Myc levels, impairs glycolysis, and compromises NK cell effector responses (Wang et al., 2024).

Dendritic cells also rely on glutamine transporters such as SLC38A2 to sustain cross-priming capacity and support CD8^+^ T cell activation within tumors (Wang et al., 2024). Therefore, tumor-driven glutamine sequestration does not merely affect tumor proliferation; it systematically reprograms immune metabolism.

### 4.2 Arginine: urea-cycle hijacking and metabolic suppression

Arginine represents another critical amino acid axis manipulated by tumors and myeloid cells. Arginine is essential for T-cell proliferation, survival, and optimal mTORC1 activation. However, tumor cells, TAMs, and MDSCs frequently express high levels of Arg1 and Arg2, enzymes that hydrolyze arginine into ornithine and urea (Kao et al., 2022; Zhang H. et al., 2024).

This enzymatic conversion produces dual immunometabolic consequences. First, extracellular arginine depletion directly impairs T-cell proliferation and effector differentiation, in part by limiting mTORC1 activation and shifting metabolic engagement away from glycolysis toward less efficient oxidative pathways (Kao et al., 2022; Zhang H. et al., 2024).

Through coordinated arginase activity and upregulation of cationic amino acid transporter, tumor and myeloid populations reshape the TME into an arginine-depleted environment that selectively disadvantages effector T cells while favoring regulatory subsets.

### 4.3 Tryptophan: IDO-driven kynurenine signaling and immune tolerance

Tryptophan catabolism represents a powerful oncometabolic pathway linking amino acid metabolism to immune tolerance. Tumor cells, TAMs, and MDSCs frequently overexpress IDO and related enzymes that convert tryptophan into kynurenine (Chapman and Chi, 2024; Kao et al., 2022; Zhang H. et al., 2024).

Tryptophan depletion has direct metabolic consequences. Because tryptophan is essential for protein synthesis and NAD^+^ biosynthesis, its depletion arrests T cells in the G1 phase of the cell cycle and can trigger Fas-mediated apoptosis (Kao et al., 2022; Zhang H. et al., 2024). In parallel, kynurenine accumulation activates the aryl hydrocarbon receptor (AhR) in T cells and DCs, promoting regulatory T cell differentiation, inducing T cell apoptosis, recruiting MDSCs, and skewing the TME toward tolerance (Chapman and Chi, 2024; Kao et al., 2022; Zhang H. et al., 2024).

In DCs, tryptophan catabolism and kynurenine signaling alter metabolic programs and cytokine production, favoring tolerogenic DC phenotypes and impairing effective T cell priming (Chapman and Chi, 2024; Zhang H. et al., 2024). The IDO–kynurenine axis, therefore, couples amino acid depletion to transcriptional and epigenetic immune reprogramming (Chapman and Chi, 2024; Kao et al., 2022; Wang et al., 2026).

### 4.4 Methionine and asparagine: epigenetic and signaling control

Methionine plays a central role in one-carbon metabolism and methylation reactions. Tumor cells overexpress methionine transporters such as SLC43A2, outcompeting T cells for methionine uptake.

Asparagine exerts additional immunometabolic effects. Asparagine depletion severely impairs CD8^+^ T-cell activation and memory formation (Kao et al., 2022; Zhang H. et al., 2024). However, tumor cells can also rely on asparagine for proliferation, complicating therapeutic modulation strategies.

Through coordinated glutamine addiction, arginine depletion, tryptophan catabolism, methionine sequestration, and asparagine competition, tumors reshape amino acid availability into a system of metabolic control. This nitrogen-centered rewiring transforms nutrient competition into a regulatory mechanism that determines immune cell activation, differentiation, survival, and exhaustion.

### 4.5 NK cells: amino acid restriction as a progressive destabilization of cytotoxic identity

NK cells rely on tightly coordinated amino acid uptake and sensing mechanisms to sustain their rapid cytotoxic responses and cytokine production. Upon activation, NK cells increase the uptake of essential amino acids, particularly leucine and arginine, to support mTORC1-dependent anabolic programming, proliferation, and production of effector cytokines such as IFN-γ and TNF-α (Yang et al., 2025). This nutrient acquisition is not passive; it is transcriptionally regulated downstream of activation signals. The heterodimeric amino acid transporter LAT1 (SLC7A5/SLC3A2) is upregulated following activation and facilitates leucine import into NK cells (Sinclair et al., 2013). IL-2 signaling further amplifies this axis by increasing SLC7A5 expression, thereby enhancing leucine uptake and reinforcing metabolic fitness in both T and NK cells (Harding et al., 2000).

Leucine is also important for sustaining NK-cell anabolic metabolism and cytotoxic activity. In the TME, reduced extracellular leucine weakens mTORC1-dependent biosynthetic programs, progressively limiting protein synthesis and impairing the replenishment of perforin, granzymes, and cytokine-related machinery. As a result, NK cells gradually lose cytotoxic efficiency and drift toward metabolic insufficiency.

Within this broader nitrogen-restricted landscape, arginine represents a second critical checkpoint. In the TME, tumor cells, tumor-associated macrophages, and MDSCs frequently express Arg1, which hydrolyzes extracellular arginine into ornithine and urea, thereby reducing local arginine bioavailability (Lemos et al., 2019; Lamas et al., 2012; Steggerda et al., 2017). Arginine deprivation impairs NK cell proliferation and significantly suppresses IFN-γ production. Notably, suppression of IFN-γ under arginine-limited conditions appears to occur predominantly through post-transcriptional mechanisms, suggesting that arginine availability regulates translational efficiency or protein stability rather than merely gene transcription (Lamas et al., 2012; Oberlies et al., 2009; Westhaver et al., 2022). In parallel, arginine scarcity destabilizes ζ-chain-mediated activation signaling, weakening signal propagation from activating receptors. Although the extent to which arginine depletion impairs degranulation remains partially debated, there is strong consensus that cytokine production and proliferative expansion are consistently compromised (Lamas et al., 2012; Westhaver et al., 2022). Importantly, arginine supplementation or pharmacologic inhibition of arginase partially restores NK functional capacity, underscoring the direct metabolic nature of this checkpoint (Westhaver et al., 2022).

A further layer of suppression is provided by tryptophan catabolism. Tryptophan metabolism introduces an additional layer of suppression through the IDO–kynurenine axis. Tumor and stromal cells frequently overexpress IDO and tryptophan-2,3-dioxygenase (TDO), catalyzing the conversion of tryptophan into kynurenine (Nachef et al., 2021; Sinclair et al., 2018). Kynurenine is transported into NK cells primarily through SLC7A5, the same transporter responsible for leucine uptake, creating a competitive intersection between nutrient import and suppressive metabolite accumulation (Della Chiesa et al., 2006; Song et al., 2011). Once internalized, kynurenine induces ROS accumulation and promotes apoptosis. It also selectively downregulates activating receptors such as NKG2D and NKp46, thereby impairing target recognition and cytotoxic engagement (Della Chiesa et al., 2006; Song et al., 2011). Pharmacologic inhibition of IDO enhances NK-mediated tumor control in preclinical models, demonstrating that tryptophan catabolism directly destabilizes NK effector stability (Wang et al., 2012).

Glutamine metabolism also intersects with NK activation, although its role differs from that in T cells. Glutamine uptake through SLC7A5 supports c-MYC expression, a transcription factor essential for anabolic growth and metabolic reprogramming in activated NK cells (Loftus et al., 2018; Ma et al., 2022). Glutamine deficiency reduces c-MYC levels, limiting NK cell expansion and effector molecule production. Interestingly, glutamine restriction does not directly collapse OXPHOS in NK cells, indicating that its primary role lies in sustaining transcriptional and biosynthetic programs rather than serving as the dominant energetic substrate (Loftus et al., 2018). Thus, glutamine functions as a signaling metabolite that stabilizes NK anabolic competence.

Serine availability further modulates NK-tumor interactions through its role in membrane lipid remodeling. Serine supports sphingomyelin biosynthesis, which is required for proper immune synapse formation and membrane protrusion architecture during target cell engagement. Serine deprivation disrupts immune synapse integrity and reduces tumor cell lysis, whereas serine supplementation or sphingomyelinase inhibition restores cytotoxic capacity (Zheng et al., 2019). In this context, serine operates as a structural determinant of synaptic stability rather than merely a metabolic substrate.

Taken together, tumor-imposed depletion of leucine, arginine, tryptophan, glutamine, and serine converges on suppression of mTORC1 activity, destabilization of c-MYC expression, accumulation of oxidative stress, downregulation of activating receptors, impairment of ζ-chain signaling, and disruption of immune synapse integrity. Rather than abruptly collapsing NK metabolism, the TME progressively destabilizes key nutrient-sensing nodes, thereby eroding NK cell cytotoxic fitness through coordinated amino acid restriction and metabolic checkpoint engagement.

### 4.6 Nitrogen on demand: amino acid adaptation and suppressive reinforcement in tumor-associated neutrophils

Amino acid metabolism represents a central and highly dynamic component of metabolic rewiring in TANs. Within the nutrient-constrained TME, TANs must continuously recalibrate their metabolic programs to sustain survival, ROS production, NET formation, and immunomodulatory activity. Rather than serving solely as substrates for protein synthesis, amino acids in neutrophils function as bioenergetic fuels, redox regulators, chromatin modifiers, and signaling intermediates that directly shape suppressive and pro-metastatic phenotypes.

Glutamine and glutamate metabolism occupy a privileged position in this rewiring. Neutrophils actively consume glutamine at high rates, converting it into glutamate via glutaminase and subsequently into α-KG, which feeds the TCA cycle and sustains OXPHOS (Lin et al., 2025; Sadiku et al., 2021). Through this pathway, glutamine-derived carbon maintains mitochondrial ATP generation and supports NADPH production, a critical determinant of ROS generation. ROS production is essential not only for microbial defense but also for neutrophil-mediated immunosuppressive activity within tumors. Glutamine is reported to be the most consumed amino acid in neutrophils, underscoring its central role in neutrophil survival under metabolic stress (Ettel and Weichhart, 2024).

Under glucose-deprived conditions typical of the TME, TANs display increased reliance on glutamate and proline catabolism to maintain mitochondrial respiration. In particular, immature low-density neutrophils utilize glutamate and proline-derived carbon to preserve oxidative metabolism and sustain NETosis, a process linked to metastatic dissemination in breast cancer models (Hsu et al., 2019). Thus, glutamine oxidation provides metabolic flexibility, allowing TANs to compensate for limited glycolytic substrates.

Tumor-derived glutamate further amplifies this metabolic program. Elevated extracellular glutamate levels promote STAT3 phosphorylation in neutrophils, driving an immunosuppressive phenotype characterized by enhanced survival and T-cell inhibitory capacity (Xiong et al., 2022). Importantly, blockade of tumor glutamate release reduces STAT3 activation and partially reverses neutrophil-mediated immunosuppression, demonstrating that glutamate functions not only as a metabolic substrate but also as a signaling molecule that stabilizes suppressive reprogramming.

As in the shared arginine-restricted environment described above, arginine metabolism constitutes another major axis of TAN-mediated immune modulation. Arginine is essential for T-cell proliferation and effector differentiation; thus, its depletion within the TME exerts direct suppressive pressure on adaptive immunity. Tumor-driven upregulation of Arg1 in neutrophils contributes to inhibition of AKT signaling in T cells, promoting cell cycle arrest and reduced viability (Tsai H. C. et al., 2023; Zhang H. et al., 2022). By depleting arginine, TANs indirectly suppress mTOR activity in T cells and impair CD8^+^ cytotoxic responses.

Arginine metabolism in neutrophils also intersects with NET formation. Peptidyl arginine deiminase 4 (PAD4) catalyzes histone citrullination, a modification required for chromatin decondensation during NETosis. Through PAD4-dependent arginine modification, neutrophils promote NET release, a process associated with tumor growth, immune evasion, and metastasis. Pharmacologic or genetic inhibition of PAD4 reduces NET formation, tumor progression, and metastatic dissemination in preclinical models (Ivey et al., 2023; Shi et al., 2020; Zhu et al., 2024). Thus, arginine metabolism in TANs integrates nutrient depletion with chromatin remodeling and pro-metastatic extracellular matrix modification.

Serine metabolism further contributes to TAN-driven tumor progression. *De novo* serine synthesis via phosphoglycerate dehydrogenase (PHGDH) supports nucleotide biosynthesis and redox homeostasis. In HCC, PHGDH activation enhances expression of chemokines such as CXCL1 and IL-8, promoting recruitment of neutrophils and macrophages and thereby amplifying tumor-supportive inflammation (Zhu et al., 2023). Through this mechanism, serine biosynthesis connects metabolic flux to chemokine-driven immune cell infiltration.

Beyond glutamine, arginine, and serine, additional amino acids contribute to neutrophil metabolic adaptation. Branched-chain amino acids (BCAAs) and glycine participate in redox regulation and biosynthetic processes by feeding shared TCA intermediates and antioxidant systems (Liu et al., 2024). These broader nitrogen fluxes underscore the competitive landscape within the TME, where tumor cells preferentially consume key amino acids such as glutamine and arginine, compelling TANs to engage alternative catabolic pathways to preserve mitochondrial integrity and suppressive function (Zhang H. et al., 2024).

Taken together, metabolic reprogramming of amino acid pathways in TANs encompasses glutamine-driven mitochondrial reinforcement, glutamate-mediated STAT3 activation, Arg1-dependent arginine depletion, PAD4-mediated chromatin modification, serine-supported chemokine production, and auxiliary nitrogen networks. These integrated circuits sustain neutrophil survival, ROS generation, immunosuppressive polarization, and metastasis-promoting functions within the TME.

### 4.7 Hijacking the nitrogen circuit: amino acid control as a driver of immune tolerance in TAMs

Amino acid metabolism in TAMs represents a deeply integrated regulatory network in which extracellular nutrient availability is continuously translated into mitochondrial dynamics, redox balance, translational control, chromatin remodeling, and immune checkpoint integration. Within the TME, TAM polarization and persistence are not governed by static M1/M2 dichotomies but by dynamic nitrogen fluxes that couple arginine, glutamine, tryptophan, BCAA, and serine metabolism to immunosuppressive plasticity. These amino acid circuits do not merely support biosynthesis; they function as metabolic switches that stabilize alternative activation programs and reinforce tumor tolerance.

Arginine metabolism constitutes one of the most decisive regulatory axes in TAM biology. The functional balance between inducible nitric oxide synthase (iNOS/NOS2) and Arg1/Arg2 activity determines whether macrophages sustain tumoricidal inflammatory programs or transition toward suppressive, pro-fibrotic phenotypes (Apiz Saab et al., 2023; Fultang et al., 2019a; Geiger et al., 2016; LaRue et al., 2022; McGinity et al., 2021; Menjivar et al., 2023; Palmieri et al., 2020; Palmieri et al., 2023; Raber et al., 2012; Reddy et al., 2023). In tumor contexts, TAMs frequently upregulate Arg1, and this enzymatic shift simultaneously depletes extracellular arginine while enhancing intracellular ornithine flux. Through increased expression of cationic amino acid transporters such as CAT-1 and CAT-2B, TAMs intensify local arginine uptake, thereby exacerbating arginine deprivation within the TME (Geiger et al., 2016; Raber et al., 2012; Rodriguez et al., 2004). Arginine scarcity not only impairs T-cell proliferation but also indirectly stabilizes macrophage suppressive programs by reducing nitric oxide production, particularly in the presence of tumor-derived asymmetric dimethylarginine (ADMA), which inhibits NOS activity and further skews macrophages toward tolerance (Chen Y. L. et al., 2022).

The conversion of arginine into ornithine feeds polyamine biosynthesis, and this metabolic branch has profound downstream consequences. Polyamines promote collagen deposition, extracellular matrix remodeling, fibrosis, and tumor growth, but they also exert intracellular effects. Increased polyamine flux supports hypusination of eukaryotic initiation factor 5A (EIF5A), a post-translational modification required for efficient translation of specific mitochondrial and metabolic transcripts. Through EIF5A hypusination, TAMs reinforce oxidative phosphorylation and TCA cycle activity, thereby stabilizing alternative activation states that rely on mitochondrial respiration rather than glycolysis (Chia et al., 2022; Puleston et al., 2019). Therapeutically, modulation of arginine metabolism (including arginine depletion, PI3Kγ inhibition to suppress ARG1 and restore NOS activity, or blockade of polyamine synthesis) has demonstrated the capacity to reprogram TAMs toward antitumor phenotypes (Fultang et al., 2019a; Kaneda et al., 2016; Miska et al., 2021; Thomas and Mattila, 2014). In this way, arginine metabolism integrates nutrient competition, translational reinforcement, mitochondrial activation, and extracellular matrix remodeling into a coherent immunosuppressive circuit.

Glutamine metabolism constitutes a second core pillar of TAM reprogramming. Within macrophages, glutamine is converted to glutamate and subsequently to α-KG, which fuels the TCA cycle and sustains mitochondrial OXPHOS (Jha et al., 2015; Palmieri et al., 2017). However, α-KG functions not only as a metabolic intermediate but also as a signaling metabolite that shapes transcriptional landscapes. Elevated α-KG supports alternative activation by promoting FAO/OXPHOS engagement, suppressing NF-κB-driven inflammatory transcription, and enabling epigenetic remodeling. As a cofactor for Jumonji domain-containing histone demethylases such as JMJD3, α-KG facilitates H3K27 demethylation and transcriptional activation of immunosuppressive genes, including Il-10, TGF-β, and Arg1 (Jha et al., 2015; Koenis et al., 2018; Liu et al., 2017; Liu et al., 2020; Liu et al., 2023; Palmieri et al., 2017). Thus, glutamine-derived carbon flux directly links mitochondrial metabolism to chromatin accessibility and suppressive gene expression. Conversely, glutamine deprivation or pharmacologic inhibition of glutaminolysis shifts macrophages toward inflammatory phenotypes characterized by increased IL-1β and TNF expression, enhancing antitumor immunity. Yet in certain tumor contexts, glutamine restriction activates HIF-1α-dependent IL-23 secretion, which promotes Treg expansion, underscoring the context-dependent outcomes of nitrogen flux perturbation (Fu Q. et al., 2019).

In parallel with the shared IDO-kynurenine framework, tryptophan metabolism further consolidates TAM-mediated tolerance. Through the expression of IDO, TAMs catalyze the conversion of tryptophan into kynurenine (Campesato et al., 2020; Mezrich et al., 2010; Munn et al., 1999). Kynurenine activates the AhR, which promotes Treg differentiation, enhances CCR2-mediated macrophage recruitment, induces CD206^+^/TGFβ^+^ immunosuppressive phenotypes, and suppresses NF-κB signaling. In this way, AhR activation integrates amino acid metabolism with immune checkpoint pathways and transcriptional reprogramming (Hezaveh et al., 2022; Labadie et al., 2019; Takenaka et al., 2019; Zhao et al., 2012). Pharmacologic blockade of AhR reshapes TAM states and enhances responsiveness to PD-1–based immunotherapies (Campesato et al., 2020), illustrating how tryptophan metabolism connects nitrogen handling to immune checkpoint integration.

Beyond these dominant axes, additional amino acid pathways broaden TAM metabolic flexibility. BCAA metabolism through BCAT1 integrates nitrogen flux with TCA cycling and redox control, influencing macrophage polarization (Fu et al., 2022). GABA signaling modulates macrophage metabolic tone and inflammatory output (Halaby and McGaha, 2021). IL-4-driven activation of the PERK–PSAT1-dependent serine biosynthesis pathway links endoplasmic reticulum stress signaling to mitochondrial function and α-KG production, further reinforcing suppressive programs (Papathanassiu et al., 2017; Pratap and Vadlamudi, 2022; Raines et al., 2022).

Single-cell and spatial transcriptomic analyses have revealed TAM subsets enriched in amino acid and purine metabolism signatures that display pro-angiogenic behavior, reduced antigen presentation capacity, and terminal differentiation phenotypes (Li et al., 2022; Ochocka et al., 2021; Raghavan et al., 2021; Zhao et al., 2021). These findings emphasize that metabolic configuration (rather than simplified polarization categories) defines TAM functional diversity within tumors.

Altogether, arginine, glutamine, tryptophan, BCAA, and serine pathways form interconnected metabolic hubs in TAMs that couple nutrient sensing to mitochondrial reinforcement, redox balance, translational control, epigenetic remodeling, and immune checkpoint signaling. Through these nitrogen-driven circuits, TAMs sustain immunosuppression, angiogenesis, fibrosis, and therapeutic resistance, positioning amino acid metabolism as a central axis of macrophage-mediated tumor immune escape (Dussold et al., 2024; Qian et al., 2024; Wang X. et al., 2025).

### 4.8 Effector T cells: amino acid competition as a progressive metabolic siege

Effector T cells enter the TME already metabolically primed for rapid expansion and cytokine production. Upon TCR engagement, they transition from a quiescent oxidative state to an anabolic program characterized by increased amino acid uptake, heightened mTORC1 signaling, enhanced glycolysis, nucleotide synthesis, and coordinated mitochondrial engagement. This shift is orchestrated transcriptionally by c-Myc and supported by HIF-1α and mTORC1-dependent translational control (Chi, 2012; Sinclair et al., 2013; Wang et al., 2011; Shen et al., 2024). Amino acid transporters are not passively expressed; they are actively induced as part of this activation program, enabling T cells to sustain the biosynthetic demands of clonal expansion and effector differentiation.

As in NK cells, leucine availability is a key determinant of anabolic fitness in effector T lymphocytes, but in T cells, this dependence is supported by more clearly defined nutrient-sensing machinery. Following activation, effector T cells strongly upregulate LAT1 (Sinclair et al., 2013). Once imported, leucine acts not only as a substrate for protein synthesis but also as a metabolic signal: intracellular leucine is sensed by Sestrin2, which interacts with the GATOR complex to regulate Rag GTPase activity. This pathway controls mTORC1 localization to the lysosomal membrane and its subsequent activation (Rashidi et al., 2020; Wolfson et al., 2016). Activated mTORC1 then drives translation of ribosomal proteins, glycolytic enzymes, and effector molecules required for cytokine production and cytotoxic function. IL-2 signaling further reinforces this circuit by increasing SLC7A5 expression, thereby amplifying leucine influx and stabilizing mTORC1 activity (Harding et al., 2000). In the TME, however, leucine availability becomes limiting, reducing mTORC1 activation and progressively impairing translational output and anabolic growth.

Methionine represents another amino acid whose depletion carries profound epigenetic consequences. Upon uptake (also largely mediated by SLC7A5), methionine is converted to SAM, the universal methyl donor required for histone and DNA methylation reactions (Roy D. G. et al., 2020). In tumor settings, cancer cells frequently overexpress SLC43A2, enabling preferential methionine uptake and depriving T cells of sufficient methionine supply (Hao M. et al., 2020; Rashkovan et al., 2022; Rodriguez et al., 2004). Reduced intracellular methionine levels in T cells lower SAM availability, leading to decreased histone H3K79 methylation. This epigenetic alteration diminishes STAT5 expression, a transcription factor critical for T-cell survival and effector differentiation. The consequence is not merely a reduction in metabolic flux but a durable change in transcriptional programming (De Martino et al., 2024; Kao et al., 2022; Wang et al., 2024). Notably, CRISPR-mediated downregulation of SLC43A2 in tumor cells restores TIL activity, demonstrating that methionine competition directly destabilizes T-cell epigenetic integrity (Huang Y. et al., 2023).

Within this metabolically restricted glutamine landscape, glutamine exerts multifaceted control over effector T-cell function. Activated T cells increase glutamine uptake through transporters such as SLC38A1 and SLC38A2, supporting nucleotide biosynthesis, lipid synthesis, and mitochondrial metabolism (Patel et al., 2019). Glutamine-derived glutamate contributes to α-KG production, feeding the TCA cycle and supporting mitochondrial function. Through GSH production, T cells maintain redox balance, buffering ROS and preserving signaling fidelity (Geiger et al., 2016). However, tumor cells frequently overexpress SLC7A11, the cystine/glutamate antiporter, thereby depleting extracellular cystine and limiting T-cell GSH synthesis (Chantranupong et al., 2016; Han et al., 2024; Liu et al., 2021a). Reduced GSH compromises redox homeostasis, promotes oxidative stress, and accelerates exhaustion. Interestingly, glutamine restriction is not uniformly suppressive. In some contexts, moderate glutamine limitation reduces terminal differentiation and promotes memory-like T-cell phenotypes (Nabe et al., 2018). Thus, glutamine flux shapes both effector intensity and differentiation trajectory.

Arginine depletion constitutes one of the most direct mechanisms of T-cell suppression in the TME. Beyond extracellular arginine depletion, downstream metabolites and intracellular arginine catabolism add further layers of suppression. Ornithine accumulation supports polyamine synthesis and further restrains CD8^+^ T-cell activation and cytotoxicity (Kao et al., 2022). In parallel, intracellular Arg2 functions as an additional metabolic checkpoint that suppresses CD8^+^ cytotoxicity, whereas its deletion enhances responsiveness to PD-1 blockade (Martí i Líndez et al., 2019). Conversely, elevated arginine levels shift metabolism toward OXPHOS and promote memory-like differentiation (Geiger et al., 2016; Wang J. et al., 2019). Arginine, therefore, operates as both a nutrient substrate and a determinant of metabolic fate.

Tryptophan metabolism further intensifies this metabolic siege. Tryptophan depletion limits protein synthesis and activates GCN2-dependent stress responses, promoting anergy, while kynurenine activates AhR, driving differentiation toward FoxP3^+^ regulatory phenotypes and suppressing CD8^+^ effector programs (Campesato et al., 2020; Munn and Mellor, 2013; Opitz et al., 2011; Qin et al., 2021). Consistent with the functional relevance of this pathway, IDO1 upregulation correlates with T-cell dysfunction and resistance to anti-PD-1 therapy (Li et al., 2018; Shu et al., 2024).

Serine and asparagine further illustrate how amino acid availability intersects with signaling. Serine supports one-carbon metabolism and nucleotide biosynthesis, sustaining proliferation (Huang et al., 2014; Ma et al., 2017). Beyond its role in protein synthesis, asparagine directly binds to the Src-family kinase LCK in CD8^+^ T cells, enhancing TCR signaling strength (Wu J. et al., 2021). Its depletion impairs activation yet may paradoxically trigger NRF2-dependent stress adaptation and enhance metabolic resilience under certain conditions (Chang et al., 2025). Methionine and cysteine exert stage-specific regulatory effects on redox balance and methylation programs (Bian et al., 2020; Chi et al., 2025; Hu et al., 2020; Srivastava et al., 2010; Wang Y. et al., 2020).

Taken together, amino acid deprivation in the TME does not represent a single bottleneck but rather a coordinated, progressive siege. Leucine scarcity dampens mTORC1 activation; methionine depletion destabilizes epigenetic programming; glutamine and cystine competition disrupt redox control; arginine depletion activates GCN2 and suppresses TCR signaling; tryptophan catabolism induces AhR-mediated tolerance; serine and asparagine fluctuations alter nucleotide synthesis and proximal TCR signaling. Through these interconnected mechanisms, the TME gradually erodes effector T-cell metabolic stability, shifting cells from expansion to dysfunction and, ultimately, toward exhaustion.

### 4.9 Thriving under nitrogen pressure: amino acid licensing and metabolic advantage in regulatory T cells

Regulatory T cells do not merely survive within the amino acid-restricted TME; they adapt to it in a manner that reinforces their suppressive function. While effector T cells progressively destabilize under nutrient competition, Tregs reconfigure their metabolic circuitry to convert amino acid flux into sustained mTORC1 signaling, redox stability, and transcriptional reinforcement of FoxP3-dependent programs. In this sense, amino acid availability does not simply sustain Tregs, it licenses their suppressive phenotype.

Amino acids such as tryptophan and arginine directly support TCR-induced mTORC1 signaling in Tregs through small GTPases, including Rag and Rheb (Shi H. et al., 2019). Unlike effector T cells, which require high-amplitude mTOR activation to sustain anabolic growth, Tregs depend on finely calibrated mTORC1 signaling to preserve lineage stability. Amino acid sufficiency maintains Rag-mediated recruitment of mTORC1 to lysosomal membranes, allowing continued integration of nutrient sensing with suppressive transcriptional programs. In this context, arginine availability does not simply sustain proliferation; it promotes IL-10 production and supports Treg maturation (Shi H. et al., 2019).

At the same time, Tregs themselves contribute to local amino acid depletion. Tregs expressing high levels of arginase can hydrolyze extracellular arginine, thereby limiting its availability to effector T cells and amplifying nutrient competition within the TME (Lowe et al., 2019; Rao et al., 2021). This creates a feedback loop in which arginine availability sustains Treg function, while Treg-mediated arginine depletion suppresses effector T-cell responses.

Glutamine and glutamate metabolism further reinforce Treg stability in tumor settings. Tumor-driven glutamine catabolism increases extracellular glutamate concentrations within the TME. In glioblastoma models, VEGF inhibition has been shown to elevate glutamate levels further, promoting Treg accumulation (Long et al., 2020). Tumor overexpression of the glutamate/cystine antiporter SLC7A11 (xCT) increases extracellular glutamate while depleting cystine, indirectly shaping the redox environment and promoting Treg induction (Long et al., 2020). Within Tregs, glutamine-derived glutamate fuels glutathione synthesis through glutamate–cysteine ligase activity, thereby maintaining intracellular redox balance and supporting FoxP3 expression and suppressive capacity (Kurniawan et al., 2020; Yan et al., 2022). By sustaining glutathione levels, Tregs preserve mitochondrial function and avoid oxidative destabilization that typically compromises effector T cells.

BCAAs contribute additional layers of metabolic regulation. BCAA metabolism influences Treg mTORC1 signaling, as branched-chain ketoacids (BCKAs) can activate mTORC1 and reinforce suppressive programs (Rao et al., 2021; Yahsi and Gunaydin, 2022). At the same time, lipid accumulation in tumor settings may interfere with BCKA catabolism, altering the balance between nutrient sensing and metabolic reinforcement. Thus, BCAA flux intersects with lipid and nitrogen metabolism to modulate Treg stability.

Within kynurenine-driven immunoregulation, tryptophan metabolism represents one of the most powerful licensed mechanisms for Treg expansion. Kynurenine promotes FoxP3^+^ Treg differentiation and immune evasion (Rao et al., 2021; Yan et al., 2022). In glioblastoma models, IDO-1 enhances Treg recruitment independently of tryptophan depletion, suggesting that kynurenine signaling itself, rather than simple amino acid starvation, drives Treg accumulation (Zhai et al., 2018). Through activation of AhR pathways, kynurenine integrates metabolic signaling with transcriptional reinforcement of regulatory phenotypes.

Methionine and cysteine further shape Treg metabolic configuration. While methionine competition can impair effector T-cell methylation programs, Tregs appear capable of sustaining sufficient SAM-dependent methylation reactions to preserve lineage stability. Cysteine availability, through its role in glutathione synthesis, supports redox control and FoxP3 stability (Yan et al., 2022). In contrast to effector T cells, which become vulnerable to oxidative stress under cystine restriction, Tregs maintain redox resilience, allowing them to persist in the same environment.

Collectively, amino acid metabolism in Tregs functions as a stabilizing architecture rather than a vulnerability. Arginine supports IL-10 production and mTORC1 signaling, while Treg-mediated arginase activity suppresses effector cells. Glutamine-derived glutamate sustains glutathione production and FoxP3 expression. BCAAs reinforce mTORC1 signaling through BCKA intermediates. Tryptophan catabolism through IDO–kynurenine signaling promotes regulatory differentiation and recruitment. Rather than succumbing to tumor-driven amino acid restriction, Tregs convert nitrogen flux into a selective advantage, preserving suppressive dominance within a metabolically constrained microenvironment (Wang R. et al., 2025; Zhang H. et al., 2024).

### 4.10 Commanding the nitrogen front: amino acid depletion as a tool of immune suppression by MDSCs

If effector T cells experience amino acid deprivation as a progressive siege, MDSCs actively orchestrate that siege. Within the TME, MDSCs do not merely respond to nitrogen scarcity; they establish it. Through coordinated regulation of arginine, tryptophan, glutamine, cystine, and related amino acid pathways, MDSCs convert nutrient depletion into a structural mechanism of immune suppression, linking extracellular amino acid depletion to intracellular stress signaling in T cells.

Arginine metabolism constitutes the most dominant suppressive axis in MDSCs. These cells upregulate Arg1 and inducible nitric oxide synthase (NOS2/iNOS), thereby diverting arginine metabolism away from supporting T-cell activation and reducing arginine bioavailability within the TME (Cimen Bozkus et al., 2015; Hu et al., 2020; Ren et al., 2023). Simultaneously, upregulation of the cationic amino acid transporter CAT2 enhances arginine uptake into MDSCs, further intensifying extracellular depletion (Cimen Bozkus et al., 2015; Ren et al., 2023). The consequences for T cells are mechanistically precise. Arginine deprivation reduces CD3ζ chain expression, impairing proximal TCR signaling and destabilizing downstream activation cascades through ZAP-70 (Moulton et al., 2015; Sun et al., 2021). At the same time, arginine scarcity activates the amino acid starvation sensor GCN2 kinase, which phosphorylates eIF2α, attenuates global translation, and enforces cell-cycle arrest (Rodriguez et al., 2007; Van De Velde et al., 2017). In parallel, mTORC1 signaling declines because arginine sensing at the Rag–Rheb axis is compromised, further limiting anabolic growth and cytokine production. NOS2 derived nitric oxide adds a further suppressive layer by promoting nitration of signaling molecules and further dampening T-cell responsiveness (Hu et al., 2020). Together, Arg1-mediated arginine depletion and NOS2-mediated signaling interference impose stress-response and nutrient-sensing checkpoints that culminate in proliferative arrest and functional paralysis of T cells.

Consistent with the shared tryptophan-restricted environment described above, MDSCs further reinforce immunosuppression by contributing to amino acid catabolism within the TME, including the establishment of tryptophan-depleted, kynurenine-rich conditions in cooperation with tumor cells and TAMs (Cao et al., 2014; Platten et al., 2019).

Glutamine metabolism further contributes to the suppressive architecture. While MDSCs consume glutamine for their own survival and metabolic activity, tumor-driven glutamine restriction more broadly reshapes immune cell differentiation. Inhibition of tumor glutamine metabolism reduces MDSC aggregation and enhances anti-tumor immunity (Bader et al., 2020; Leone et al., 2019; Ron-Harel et al., 2016; Tombari et al., 2023). This suggests that glutamine flux within the TME influences MDSC accumulation and persistence. Additionally, glutamine-derived metabolites participate in redox regulation and support mitochondrial stability in suppressive myeloid populations.

Cystine metabolism intersects with glutamine flux through the SLC7A11 transporter, which exchanges intracellular glutamate for extracellular cystine. Tumor overexpression of SLC7A11 depletes cystine from the TME, impairing glutathione synthesis in T cells and promoting oxidative stress–induced dysfunction (Chantranupong et al., 2016; Han et al., 2024; Liu et al., 2021a). Although SLC7A11 is primarily described in tumor cells, the redox consequences of cystine depletion reinforce the suppressive landscape in which MDSCs operate.

Methionine competition also influences immune balance. Tumor-mediated methionine depletion alters methylation capacity in T cells, indirectly favoring suppressive myeloid dominance (Hao M. et al., 2020; Huang X. et al., 2023). As methylation reactions regulate transcriptional programs governing activation and exhaustion, methionine scarcity contributes to long-term epigenetic destabilization of effector responses.

Thus, MDSCs enforce amino acid-dependent immune suppression through coordinated arginine drainage, nitric oxide production, tryptophan catabolism, glutamine redistribution, and redox modulation. Rather than relying on a single suppressive mediator, MDSCs integrate multiple amino acid circuits to create a metabolically restrictive environment in which adaptive immunity becomes progressively arrested.

Through this multilayered nitrogen control, MDSCs function as metabolic gatekeepers of tumor immune escape, converting amino acid flux into sustained suppression and therapeutic resistance.

## 5 Arsenal of intervention: therapeutic targeting of metabolism to rewire anti-tumor immunity

Metabolic pressure in the TME is increasingly treated as a therapeutic variable that can be pharmacologically tuned to constrain tumor fitness while enabling anti-tumor immunity. The sections below summarize intervention strategies that target nutrient utilization, metabolite stress, and upstream sensing pathways to improve immunotherapy performance without broadly disabling immune-cell metabolism.

### 5.1 Cut the sugar and clear the acid: targeting glycolysis and lactate burden

Pharmacologic control of glucose-driven immunosuppression in cancer is increasingly approached as a two-sided problem: (i) restraining tumor and stromal glucose utilization and downstream lactate stress, while (ii) preserving, or even re-enabling, immune-cell metabolic programs required for cytotoxic function. Immune checkpoint pathways, particularly inhibitory receptor-ligand signals such as PD-1/PD-L1 and CTLA-4 that regulate T-cell activation and metabolism, operate directly at this metabolic-immune interface. PD-1 signaling inhibits PI3K and mTORC1, limiting anabolic metabolism, including glycolysis (Zhang J. et al., 2024), and can be induced by lactate (Kumagai et al., 2022), whereas CTLA-4 suppresses effector T-cell glycolysis and supports Treg stability (Frauwirth et al., 2002; Zappasodi et al., 2021); thus, blocking these checkpoints has direct metabolic effects, not only immune-signaling effects (Morad et al., 2021). At the same time, checkpoint molecules also shape metabolism more directly. Some checkpoint ligands expressed by tumor cells, such as PD-L1 and B7-H3, can promote aerobic glycolysis through the PI3K–AKT–mTOR pathway (Lim et al., 2016), which may strengthen tumor glucose utilization. In parallel, inhibitory receptors on T cells beyond PD-1 and CTLA-4, including Tim-3 and LAG-3, can further reduce glycolysis in immune cells (Ferris et al., 2014; Li et al., 2019). For example, Tim-3 has been reported to suppress glycolysis in Jurkat T cells by reducing GLUT1 expression (Lee et al., 2020). These links explain why metabolic drugs and immune checkpoint inhibitors (ICIs) are often positioned as complementary levers but also why the timing, delivery, and selectivity of metabolic inhibition matter: reducing cancer glycolysis can limit proliferation yet may also blunt effector immunity in some settings (Bonuccelli et al., 2010; Sukumar et al., 2013; Kouidhi et al., 2018).

An initial therapeutic strategy is to limit tumor glucose availability or interfere with glucose uptake mechanisms. Targeting glucose supply in hypoxic lesions is described as a viable strategy to weaken tumor cells in nutrient- and oxygen-limited tumor regions (Hensley et al., 2016). On the uptake side, direct inhibition of glucose transport is exemplified by a GLUT1-directed strategy: TH-G313B is reported to target GLUT1 and reduce glucose uptake and proliferation (Gao et al., 2020). Because T cells also depend on glucose, the goal is usually not to deprive the whole TME of glucose, but to limit glucose uptake by tumor cells more selectively so that antitumor lymphocytes can access more nutrients. In this scenario, nanomedicine extends glucose-targeting strategies by delivering metabolic enzymes directly into tumors to deplete nutrients locally. In this context, glucose oxidase (GOx) is used to consume glucose *in situ* by converting it into gluconic acid, thereby lowering glucose availability and reducing lactate/ATP production (Fu L. H. et al., 2019; Xie et al., 2019). However, GOx has important limitations, including poor bioavailability, rapid inactivation, and systemic toxicity risks such as hypoglycemia and organ damage (Hao et al., 2019; Zhou et al., 2020). For this reason, nanoparticle delivery is used to localize GOx activity in tumors and improve efficacy while reducing systemic toxicity (Gao F. et al., 2019; Xie et al., 2019).

HK2 is repeatedly highlighted as a key target because it controls glycolytic flux at the first step of the pathway. Mechanistically, HK2 catalyzes the conversion of glucose to glucose-6-phosphate (Tan and Miyamoto, 2015), and elevated HK2 activity is associated with high metabolic demand and rapid growth in some tumors (Feng et al., 2020). HK2 depletion in glioblastoma reduced proliferation and angiogenesis, with lower HIF-1α and VEGF, but it also increased tumor invasion (Wolf et al., 2011). This suggests that inhibiting glycolysis can suppress tumor growth programs while simultaneously favoring adaptive invasive behavior, supporting the use of combination strategies rather than HK2-targeted monotherapy. Within this context, 2-DG enters cells through glucose transporters and is then phosphorylated by HK2 to 2-DG-6-P. Because this metabolite cannot be converted to fructose-6-P, it accumulates inside cells and inhibits tumor glucose metabolism (Jin et al., 2016). Preclinical studies also use 2-DG as a chemotherapy adjuvant (Emmett et al., 2021; Mena et al., 2022; Karbhari et al., 2023), with reported benefits in combination with gefitinib (Kim et al., 2020), doxorubicin (Ma et al., 2015), and cisplatin (Simons et al., 2007). This strategy has also reached clinical testing, including in advanced solid tumors (NCT00096707 and NCT00633087), where tolerability was reported (Stein et al., 2010; Wang X. et al., 2025). In addition to their use as free drugs, glycolysis inhibitors such as 2-DG and lonidamine are also incorporated into nanocarrier platforms, mainly to improve tumor delivery and reduce systemic exposure (Luo et al., 2021; Zhao L. P. et al., 2022; Li T. et al., 2023). More broadly, these nanoplatforms are designed not only to impose metabolic stress on tumor cells but also to modulate the microenvironment in ways that support T-cell infiltration and function. In HCC, benitrobenrazide (BNBZ) is described alongside 2-DG as inhibiting glycolysis *in vivo* and *in vitro*, with oral BNBZ significantly inhibiting tumor growth (Broecker-Preuss et al., 2017; Zheng et al., 2021). In the same intervention tier, upstream regulators and contextual dependencies are acknowledged: inhibiting glycolytic nodes, transporters, HIF-1α, and mTOR is listed as part of this strategy class (Chen et al., 2021; Stine et al., 2021; Yang S. et al., 2022).

PFKFB3 is positioned as another actionable control point that affects glycolytic rate and lactate output, as its overexpression is described in aggressive tumors (Bartrons et al., 2018). A strategy to reduce tumor acidity combines 3PO (a PFKFB3 inhibitor) with lactate oxidase (LOx) in HMnO_2_ (hollow manganese dioxide) nanoparticles (Gao F. et al., 2019). This approach is designed to lower glycolytic output (including lactate and ATP production) while simultaneously consuming lactate, with the goal of reducing acid stress in the TME and improving the response to IC-blockade (ICB) by reversing immunosuppressive features of the tumor immune microenvironment (TIME) (Jiang et al., 2025). A similar combination logic is also seen in checkpoint-based regimens: PFK-158 (another PFKFB3 inhibitor) combined with anti-CTLA-4 improved responses in B16 melanoma (Chesney et al., 2016). Together, these approaches follow the same principle: first reduce tumor glycolytic- and lactate-driven suppression and then use immunotherapy in a metabolically less hostile microenvironment.

LDH levels correlate positively with tumor size and clinical severity (Girgis et al., 2014; Sun et al., 2014). Pharmacologic or siRNA-mediated LDH inhibition can reduce intratumoral lactate and promote tumor regression (de la Cruz-López et al., 2019), although the immune effects are not uniform and may include reduced T-cell numbers and IFN-γ production in some settings (Kouidhi et al., 2018). In one experimental setting, LDHA inhibition reduced tumor glucose use and lactate production, creating a higher-glucose/lower-lactate environment that supported CD8^+^ TIL effector function while destabilizing Tregs (Yan J. et al., 2023). In the same setting, CTLA-4 blockade also contributed to Tregs’ destabilization through effects on glucose metabolism, providing a rationale for combining LDHA inhibition with anti-CTLA-4 to preserve nutrients for CD8^+^ TILs. A metal-phenolic nanomedicine carrying GSK2837808A was used to implement this strategy by suppressing aerobic glycolysis and establishing the high-glucose/low-lactate condition (Yan J. et al., 2023). Reported readouts included reduced extracellular acidification rate and changes in extracellular glucose and lactate levels, together with higher glucose uptake by adjacent CD8^+^ T cells in co-culture and reduced lactate uptake in Tregs cells, consistent with glucose/lactate-dependent regulation of IFN-γ output (Yan J. et al., 2023). Combination data further support this axis: oxamate (an LDHA inhibitor) plus pembrolizumab increased activated CD8^+^ T-cell infiltration and improved pembrolizumab efficacy) (Qiao et al., 2021).

Another pharmacologic strategy is to block lactate transport rather than lactate production. This transport is mainly mediated by monocarboxylate transporters (MCTs), especially MCT1 and MCT4. By inhibiting lactate export/import, lactate can accumulate inside tumor cells, which slows glycolysis and can promote cancer cell death (de la Cruz-López et al., 2019). This approach can also support anti-tumor immunity, as lactate transport inhibition has been associated with increased IL-2 and IFN-g secretion in T cells (Chirasani et al., 2012) and with reduced tumor proliferation together with improved T-cell activation (Kouidhi et al., 2018). Because MCT1 and MCT4 can compensate for each other, dual-targeting strategies are often needed. In this context, syrosingopine plus metformin has been shown to induce a lethal energy crisis through combined MCT1/4 inhibition (Benjamin et al., 2018). Combination logic also extends to immunotherapy: AZD3965 (an MCT1 inhibitor) improved anti-tumor responses when combined with anti-PD-1 treatment (Jiang et al., 2020; Huang T. et al., 2021). A related nanomedicine approach builds on the same biology, since MCT4-mediated lactate efflux and MCT1-mediated lactate influx help maintain metabolic symbiosis between hypoxic and oxygenated tumor regions (Payen et al., 2020; Sun et al., 2020). In that setting, nanoparticle delivery of AZD3965 improved CD8^+^ T-cell responses when paired with anti-PD-1 (Payen et al., 2020), although this strategy may require careful targeting because MCTs are also expressed by normal cells, including infiltrating CD8^+^ T cells (Fischer et al., 2007).

Because lactate-driven acidosis is itself immunosuppressive, several interventions focus on neutralizing acidity or exploiting pH selectivity. Oral bicarbonate is described to inhibit tumor growth when combined with anti-PD-1 in melanoma and to improve survival when combined with adoptive T cell transfer (Koltai, 2016; Kouidhi et al., 2018). Similarly, bicarbonate-based neutralization of tumor acidity can increase T-cell infiltration and enhance anti-tumor immunity, including in settings combined with checkpoint blockade and adoptive cell transfer (ACT) (Pilon-Thomas et al., 2016). Additional pH-directed strategies use antibodies that act preferentially under acidic conditions, including antibodies targeting VISTA (V-domain Ig suppressor of T-cell activation) or its receptor PSGL-1 (P-selectin glycoprotein ligand-1), which can reverse immunosuppression and promote tumor rejection in the MC38 model (Johnston et al., 2019). Acidosis is also linked to functional immune rewiring: low pH can promote monocyte-derived DC phenotypes with reduced glucose consumption and inhibited mTORC1 signaling (Erra Díaz et al., 2020). This provides an additional pharmacologic rationale for buffering acidity or controlling lactate, not only as tumor-targeting strategies but also as measures that help restore immune function.

A common theme across these strategies is that metabolic targeting and immunotherapy work best when used together. Metabolic interventions can reduce tumor metabolic dominance and make the TME less suppressive, while immunotherapy can then take advantage of this more permissive setting. This logic is reflected in several combinations already mentioned, including PFK-158 plus anti-CTLA-4, oxamate plus pembrolizumab, AZD3965 plus anti-PD-1, and bicarbonate plus anti-PD-1 or ACT (Bader et al., 2020). The same principle also applies to immune co-stimulation, which can help sustain T-cell metabolism when tumor-focused metabolic drugs risk suppressing glycolysis more broadly. In this context, CD28 enhances aerobic glycolysis and mitochondrial fusion (Jacobs et al., 2008), ICOS (inducible T-cell co-stimulator) increases glycolysis through the PI3K–AKT–mTOR pathway (Gigoux et al., 2014; Zeng et al., 2016), and 4-1BB, OX40, and GITR (co-stimulatory receptors in the TNF receptor family) increase glucose uptake in CD8^+^ T cells (Choi et al., 2016; Tsurutani et al., 2016; Sabharwal et al., 2018; Kim et al., 2020).

An additional and underexplored dimension of this metabolic-immunotherapy interface concerns DCs as both targets of tumor-imposed metabolic suppression and actionable nodes for therapeutic enhancement. The glycolytic, hypoxic, and lactate-rich TME established by tumor cells directly impairs DC function, converting immunogenic antigen-presenting cells into tolerogenic regulators through glycolytic stress, lactate signaling, hypoxia, and adenosine accumulation. Restoring DCs metabolic fitness and functional competence, therefore, represents a complementary strategy alongside the metabolic interventions discussed above. *In vivo* DC modulation approaches include expansion of DC progenitors with FLT3L or GM-CSF, activation with TLR agonists or CD40 agonists to enhance IL-12-producing DCs populations and sensitize tumors to PD-1 blockade, and engineering of DCs to secrete CXCL9 and CXCL10, which attract CXCR3^+^ T cells and overcome ICB resistance in preclinical lung cancer models (Marciscano and Anandasabapathy, 2021; Garris et al., 2018; Lim et al., 2024). Furthermore, targeting IL-4 signaling or the AXL receptor in mregDC subsets has been proposed to restore cDC1 functionality and IL-12 production (Maier and Leader, 2020; Moon et al., 2025). These approaches are conceptually aligned with the broader principle articulated in this section: metabolic decompression of the TME creates a more permissive environment in which DC-targeted interventions can more effectively prime and sustain antitumor T-cell responses.

Together, these findings (summarized in Table 1) support a practical combination strategy: pair tumor-directed metabolic inhibition with checkpoint blockade or co-stimulatory signals to preserve effector T-cell metabolism and function.

**TABLE 1:** Glucose-lactate axis. Summary of drug-based interventions targeting the glucose–lactate axis, including combination settings, primary metabolic effects, and outcomes.

Drug	Combination therapy	Primary metabolic effect	Outcome	Key references
TH-G313B	—	Decreases GLUT1-mediated glucose uptake	Decreases glucose uptake and proliferation	Gao et al. (2020)
Glucose oxidase (GOx) (nanodelivery platform)	—	Glucose → gluconic acid → decreases glycolysis and decreases lactate/ATP	Local ‘starvation’ and decreases lactate/ATP; systemic toxicity risk motivates localization	Fu L. H. et al. (2019), Xie et al. (2019), Hao et al. (2019), Zhou et al. (2020), Gao F. et al. (2019)
2-DG	Chemotherapy adjuvant: cisplatin/doxorubicin/gefitinib	HK2-linked glycolysis blockade	Decreases tumor glycolysis; tolerability noted	Jin et al. (2016), Stein et al. (2010), Wang X. et al. (2025), Emmett et al. (2021), Mena et al. (2022), Karbhari et al. (2023), Simons et al. (2007), Ma et al. (2015), Kim et al. (2020), NCT00096707, NCT00633087
Lonidamine (nanocarriers)	—	Decreases aerobic glycolysis via HK2/mitochondrial effects	Decreases glycolysis (delivery-optimized in nanocarriers)	Luo et al. (2021), Zhao L. P. et al. (2022), Li B. et al. (2023)
Benitrobenrazide (BNBZ)	±2-DG	Decreases glycolysis (HCC context)	Inhibition of tumor growth	Broecker-Preuss et al. (2017), Zheng et al. (2021)
3PO/PFK15	—	PFKFB3 inhibition → decreases lactate output	Decreases lactate-driven suppression	Bartrons et al. (2018), Gao F. et al., (2019)
Lactate oxidase (LOx)	ICB (with 3PO, HMnO2 NPs)	Increases lactate consumption (plus glycolysis inhibition from 3PO)	Improved ICB by reversing TIME features	Gao F. et al. (2019), Jiang et al. (2025)
PFK-158	Anti-CTLA-4	PFKFB3 inhibition → decreases glycolytic rate/lactate	Improved responses in B16 model	Chesney et al. (2016)
GSK2837808A (LDHA inhibitor)	± anti-CTLA-4	Decreases lactate; high-glucose/low-lactate shift	ECAR decreases; extracellular glucose/lactate shifts; increases CD8 glucose uptake; decreases Treg lactate uptake	Yan J. et al. (2023)
Oxamate (LDHA inhibitor)	Pembrolizumab	Decreases lactate	Increases activated CD8 infiltration and pembrolizumab efficacy	Qiao et al. (2021)
AZD3965 (MCT1 inhibitor)	Anti-PD-1	Lactate trapping via MCT1 blockade	Improved anti-tumor responses with anti-PD-1	Jiang et al. (2020), Huang T. et al. (2021)
Syrosingopine + metformin	​	Dual MCT1/4 blockade → energy crisis	Lethal energy crisis	Benjamin et al. (2018)
Bicarbonate	Anti-PD-1 and/or ACT	Decreases acidosis (buffering)	Inhibited tumor growth with anti-PD-1; improved survival with ACT; increases T-cell infiltration	Koltai (2016), Kouidhi et al. (2018), Pilon-Thomas et al. (2016)
VISTA/PSGL-1 antibodies (acidic pH-selective)	± PD-1 blockade	pH-selective checkpoint targeting	Reversed immunosuppression; MC38 rejection with PD-1 co-blockade	Johnston et al. (2019)

### 5.2 Rationing the fat: targeting lipid metabolism without starving immunity

Therapeutic modulation of lipid metabolism in cancer has increasingly converged on drugging lipid synthesis, lipid uptake/handling, and lipid utilization pathways with the dual intent of (i) restricting tumor-supportive lipid programs and (ii) reversing lipid-driven immune dysfunction in the TME. A central pharmacologic node is the SREBP-FASN lipogenic axis, where inhibition of lipid metabolism-related proteins/enzymes is highlighted as a strategy to suppress tumor growth and counter metabolic adaptability. Consistently, SREBP suppression can inhibit tumor growth and promote cancer cell death, supporting the broader use of small-molecule strategies targeting FASN (Flavin et al., 2011). In FASN-dependent HCC, the FASN inhibitor TVB-3664 significantly reduced tumor growth (Wang H. et al., 2022). More broadly, many tumors sustain their high lipid demand by increasing uptake of exogenous lipids and/or activating lipid and cholesterol biosynthesis pathways, and lipid droplet accumulation correlates with aggressiveness (Beloribi-Djefaflia et al., 2016; Wegiel et al., 2018). These features support direct targeting of FA synthesis, particularly through FASN inhibition (Mullen and Yet, 2015; Lu T. et al., 2018). Additional FASN-targeting combination development includes TVB-2640 with paclitaxel and trastuzumab in metastatic HER2-positive breast cancer (NCT03179904) and TAS-118 plus oxaliplatin versus S-1 plus cisplatin in advanced gastric cancer (NCT02322593), where TAS-118 + oxaliplatin demonstrated a clinically meaningful improvement in efficacy (Kang et al., 2020).

A second major pharmacological objective is correcting lipid-induced immune impairment, particularly in antigen presentation and suppressive myeloid programs. Intracellular lipid accumulation in myeloid cells is described to enhance oxidative metabolism and promote immunosuppressive function (Le Bourgeois et al., 2018), and exposure to oleate can polarize bone marrow-derived myeloid cells toward an M2-like TAM phenotype (Wu et al., 2019), supporting approaches that either reduce lipid loading or disrupt lipid-fueled suppressive phenotypes. Lipid accumulation in tumor-infiltrating DCs is linked to defective antigen presentation (Herber et al., 2010), and in an ovarian cancer model, DCs acquired excess lipids exogenously from the TME with poor T cell priming and activation (Jiang et al., 2018). Pharmacologic reduction of lipid abundance in DCs is, therefore, presented as an immune-restorative intervention: inhibiting FA synthesis with the ACC inhibitor TOFA normalized DC lipid levels and substantially improved a cancer vaccine response (Herber et al., 2010), while direct FASN inhibition partially rescued tumor-infiltrating DC function (Jiang et al., 2018). Lipid metabolism is also tied to immunosuppressive cell maturation and function: immunosuppressive populations (TAM-M2, Tregs, and MDSCs) are described as relatively more FAO-dependent than glycolysis (Wang et al., 2017). Increased FASN is described as contributing to the functional maturation of Tregs and TAM-M2 cells, and FASN inhibition impairs TAM tumor-promoting effects by suppressing TNF-α, IL-6, IL-10, and ROS (Lim et al., 2021b). Lipid-metabolism targeting can also support T-cell persistence under repeated stimulation: FASN inhibition in primary human T cells (using C75 or siRNA knockdown) protected T cells from restimulation-induced cell death by reducing induction of FAS ligand (FASL) (Voss et al., 2019). Consistently, inhibiting FAO is included among metabolic adjustments that can improve T-cell anti-tumor effects and reduce tumor progression (Varghese et al., 2021; Wang J. et al., 2021).

Lipid accumulation in tumor-infiltrating DCs is not merely a metabolic epiphenomenon but a direct impairment of antigen cross-presentation, as documented in patients with advanced lung cancer (Arai et al., 2018). DC vaccines (DCVs) offer a strategy to circumvent intratumoral DC dysfunction by generating and activating DCs *ex vivo*; although safe and immunogenic across multiple solid tumor types, their standalone clinical efficacy has been modest, and optimization of antigen loading, DCs subsets, maturation protocols, and patient selection criteria remains an active area of development (Hato et al., 2024; Gardner et al., 2020; Wculek et al., 2019; Wang Y. et al., 2020; Zanotta et al., 2024; Amon et al., 2020). Critically, combining DCVs with CTLA-4 or PD-1 blockade has demonstrated the capacity to increase response rates and convert immunologically “cold” tumors into T-cell-inflamed lesions (Hato et al., 2024; Gardner et al., 2020; Garris and Luke, 2020; Zanotta et al., 2024; Amon et al., 2020). These combination strategies align directly with the lipid-targeting interventions described above: pharmacological normalization of DC lipid content through ACC or FASN inhibition may enhance the immunogenicity of both endogenous and vaccine-derived DCs, providing a mechanistic rationale for integrating lipid-metabolism inhibitors into DCV-based immunotherapy regimens.

A third cluster of interventions targets lipid uptake/trafficking and cholesterol handling to directly enhance T cell performance and synergize with ICIs. CD36 is described as a mediator of intracellular FA uptake and lipid droplet growth (Su and Abumrad, 2009; Pepino et al., 2014). Its plasma-membrane localization and FA binding require DHHC4-mediated palmitoylation, and depalmitoylation is required for intracellular transport to endocytosis (Wang J. et al., 2019; Hao J. W. et al., 2020). In tumors, CD36 and FABP5 are described as promoting Treg survival and function (Field et al., 2020; Wang H. et al., 2020). In CD8^+^ TILs, CD36-mediated FA uptake is linked to lipid peroxidation and ferroptosis, while CD36 deficiency inhibited tumor growth (Ma et al., 2021). In addition, OxLDL uptake through CD36 on CD8^+^ TILs induced lipid peroxidation and promoted intratumoral CD8^+^ T cell dysfunction (Xu S. et al., 2021). These findings support lipid-uptake blockade as an immunometabolic strategy, especially to limit lipid peroxidation and suppress lipid handling. Consistently, DC accumulation of oxidized lipids (including triacylglycerols) is associated with defective antigen presentation (Veglia and Gabrilovich, 2017; Arai et al., 2018), and electrophilic oxidized lipids can impair cross-presentation by binding HSP70 and blocking peptide–MHC-I transport to the cell surface (Veglia et al., 2017).

Cholesterol esterification is another pharmacologic target, since cholesterol metabolism directly regulates CD8^+^ T-cell anti-tumor activity. Inhibiting ACAT1 increases plasma-membrane cholesterol and enhances CD8^+^ T-cell proliferation and effector function (Yang et al., 2016). This also translates into a combination benefit as ACAT1 inhibition plus anti-PD-1 improved anti-tumor efficacy (Patsoukis et al., 2015). Avasimibe (a sterol O-acyltransferase 1/ACAT1 inhibitor) similarly increases cholesterol levels, improves T-cell effector function and proliferation, and cooperates with PD-1 inhibitors in melanoma eradication *in vivo*. It also shows anti-tumor activity in mouse models, particularly in combination with anti-PD-1 (Yang et al., 2016). Beyond cholesterol esterification, effector T-cell lipid metabolism can also be pharmacologically reprogrammed by inhibiting group IVA phospholipase A2 (cytosolic phospholipase A2α, cPLA2α), which improved anti-tumor immunity and immunotherapy efficacy in tumor-bearing mouse models (Liu et al., 2021c). Additional immunometabolic combination agents include celecoxib (a COX-2 inhibitor) and E7046 (an EP4 receptor antagonist); together with avasimibe, these agents produced synergistic anti-tumor immune responses when combined with PD-1 or CTLA-4 inhibitors in mouse tumor models (Li et al., 2016; Yang et al., 2016; Albu et al., 2017).

An emerging dimension of lipid-targeting strategies concerns the selective modulation of ferroptosis across immune and tumor cell populations. As described in the lipid sections above, CD36-mediated uptake of oxidized lipids drives lipid peroxidation and ferroptotic death in intratumoral CD8^+^ T cells, impairing their cytotoxic function and limiting responses to checkpoint blockade (Ma et al., 2021; Xu S. et al., 2021). Genetic ablation of Cd36 in effector CD8^+^ T cells restores cytokine production, enhances tumor control, and synergizes with anti-PD-1 therapy (Ma et al., 2021). Pharmacologic ferroptosis inhibitors applied to CD8^+^ T cells similarly rescue antitumor activity and potentiate ICB efficacy (Gong et al., 2022; Liang et al., 2025). A complementary strategy addresses the upstream driver: cystine supplementation or tumor SLC7A11 deletion restores glutathione synthesis, prevents CD36-driven ferroptosis in T cells, and improves antitumor responses (Han et al., 2024). Critically, the therapeutic goal is not to block ferroptosis globally but to maintain the asymmetry whereby tumor cells undergo ferroptosis while CD8^+^ T cells are protected. This balance has been achieved in preclinical settings using albumin nanoparticles co-loaded with a CD36 inhibitor and a ferroptosis inducer, which enhanced tumor ferroptosis while suppressing lipid peroxidation in T cells and reducing MDSCs (Yang et al., 2024). On the Treg side, Treg-specific deletion of Gpx4 sensitizes activated Tregs to ferroptotic death, reduces their intratumoral survival, and enhances antitumor immune responses in mouse models (Xu C. et al., 2021); however, as GPX4 loss also damages effector T cells, any therapeutic GPX4 targeting would require Treg-specific or tumor-specific delivery to avoid blunting overall immunity (Klopotowska et al., 2025). Together, these findings position the CD36–GPX4–SLC7A11 axis as a tractable therapeutic node within lipid metabolism, with the potential to shift the ferroptosis balance in favor of antitumor immunity when combined with ICIs.

Collectively, these pharmacologic strategies define a coherent therapeutic playbook, block lipogenesis (SREBP/FASN), normalize lipid-driven antigen-presentation defects (ACC/FASN), disrupt suppressive lipid handling (CD36/oxidized lipids), and rewire cholesterol and lipid signaling to potentiate ICIs (ACAT1/avasimibe; COX2/EP4; group IVA phospholipase A2), with the strongest effects frequently emerging when lipid-metabolism interventions are integrated into rational immunotherapy combinations (Herber et al., 2010; Yang et al., 2016; Jiang et al., 2018). Overall, lipid-directed therapies converge on suppressing lipogenesis, limiting lipid uptake and oxidized-lipid stress, and reprogramming cholesterol handling to restore antigen presentation and strengthen T-cell function, with several agents showing synergy with ICIs; the main targets, mechanisms, and reported outcomes are summarized in Table 2.

**TABLE 2:** Lipid metabolism. Summary of lipid-directed therapeutic agents, including their combination use, primary metabolic effects, and outcomes.

Drug	Combination therapy	Primary metabolic effect	Outcome	Key references
TVB3664 (FASN inhibitor)	—	Decreases fatty acid synthesis	Significantly reduced tumor growth (FASN-dependent HCC)	Wang H. et al. (2022)
TVB-2640 (FASN axis)	Paclitaxel/trastuzumab (trial)	Decreases lipogenesis	Tumor growth limitation	NCT03179904
TAS-118	Oxaliplatin	​	Clinically meaningful improvement in efficacy	Kang et al. (2020), NCT02322593
TOFA (ACC inhibitor)	Cancer vaccine	Decreases DC lipid overload	Normalized DC lipids; substantially enhanced vaccine response	Herber et al. (2010)
Avasimibe (ACAT1 inhibitor)	Anti-PD-1	Increases membrane cholesterol (esterification blockade)	Improved CD8 function and synergy with anti-PD-1	Yang et al. (2016), Patsoukis et al. (2015)
Celecoxib (COX-2 inhibitor)	PD-1 or CTLA-4 inhibitors	Modulates lipid mediator signaling	Synergistic anti-tumor immune responses (models)	Li et al. (2016), Yang et al. (2016), Albu et al. (2017)
E7046 (EP4 antagonist)	PD-1 or CTLA-4 inhibitors	Modulates prostaglandin signaling	Synergistic anti-tumor immune responses (models)	Li et al. (2016), Yang et al. (2016), Albu et al. (2017)
C75 (FASN inhibitor)	—	Decreases fatty acid synthesis (T cells)	Protected T cells from restimulation-induced cell death via decreases FASL	Voss et al. (2019)
CD36 inhibitor + ferroptosis inducer (LHS albumin NPs)	—	Bidirectional ferroptosis modulation: Induces tumor ferroptosis and protects T cells	↑ CD4/CD8, ↓ MDSCs/Tregs; ICB-like immune remodeling (TNBC)	Yang et al. (2024)
Ferroptosis inhibitors (e.g., ferrostatin-1)	Anti-PD-1	Blocks lipid peroxidation in CD8+ T cells	Rescues antitumor activity; potentiates ICB	Ma et al. (2021) , Gong et al. (2022), Liang et al. (2025)
Gclc overexpression/cystine supplementation	—	Restores GSH synthesis; prevents CD36-driven ferroptosis in T cells	Improved antitumor T-cell responses	Han et al. (2024)
Treg-specific GPX4 targeting	—	Sensitizes Tregs to ferroptotic death	↓ intratumoral Tregs; ↑ antitumor immunity (mouse models)	Xu C. et al. (2021), Klopotowska et al. (2025)

### 5.3 Breaking bad habits: drugging glutamine, arginine, tryptophan, and oncometabolites

Therapeutic targeting of amino acid metabolism is increasingly used to modulate both tumor metabolism and the immune compartment, with the goal of reducing nutrient-driven immune suppression and improving the efficacy of ICIs. In practice, this includes four main strategies: inhibiting key metabolic enzymes involved in tumor growth or immunosuppressive signaling, blocking amino acid transport to increase nutrient availability for immune effector cells, depleting specific amino acids in tumors with auxotrophic vulnerabilities, and combining these approaches with ICIs or conventional therapies to limit metabolic escape.

A major therapeutic axis is glutamine utilization, where drug development focuses on suppressing glutaminolysis to constrain tumor growth while shifting nutrient availability within the TME in favor of antitumor immunity. CB-839 is a selective GLS1 inhibitor for glutamate/glutamine-dependent cancers, with the therapeutic goal of disrupting glutamate metabolism and limiting production of proliferative intermediates (Yang W. H. et al., 2021). It has entered dose-escalation clinical testing with safety, pharmacokinetic/pharmacodynamic, and biomarker endpoints (NCT02071862), and it has also been used in combination settings, including enhancement of cisplatin-induced apoptosis in triple-negative breast cancer models (Wang X. et al., 2025). In immunotherapy-oriented strategies, CB-839 combined with anti-PD-1 or anti-CTLA-4 antibodies increased effector T-cell infiltration and improved checkpoint inhibitor activity in mouse melanoma models (Varghese et al., 2021). Additional glutamine/glutamate-targeted strategies use different levels of selectivity to restrict tumor nutrient use. Trigriluzole (BHV-4157) is being tested as a glutamate-deprivation approach in combination with nivolumab or pembrolizumab (NCT03229278) (Silk et al., 2020). At the other end of the spectrum, DON (6-diazo-5-oxo-L-norleucine) covalently targets multiple glutamine-metabolizing enzymes, although this broader activity creates challenges for precision use and resistance management (Lemberg et al., 2018). A more global approach has also been tested by combining broad glutamine metabolism blockade with anti-PD-1, which markedly improved antitumor effects while suppressing tumor metabolism and promoting T-cell glucose metabolism, epigenetic reprogramming, and cytotoxic function (Leone et al., 2019). Overall, these strategies aim to limit tumor glutamine use while preserving or strengthening anti-tumor immune function, especially in combination with ICIs.

Arginine metabolism is treated as a second high-value target space because pharmacologic control of arginine availability can be used either to exploit tumor dependence or to reverse myeloid-driven immune suppression. On the tumor-directed side, malignant melanoma and HCC are reported as more susceptible to arginine deprivation therapy using arginine-degrading enzymes (Zou et al., 2019). This deprivation strategy is translated into ICI combinations, including ADI-PEG20 (a PEGylated arginine deiminase enzyme that depletes extracellular arginine) with pembrolizumab in advanced solid cancers (NCT03254732). A more selective approach targets arginine uptake rather than systemic depletion: selective inhibition of arginine transport reduces arginine uptake by HCC cells and increases arginine availability for anti-tumor immune cells (Zhang J. et al., 2024). On the immune-conditioning side, arginase inhibition is used to restore local arginine levels and reduce immunosuppression. In this setting, arginase blockade has been associated with restored arginine availability, tumor regression, and improved T-cell function (Miret et al., 2019). A leading example is CB-1158, which blocks myeloid cell-mediated immune suppression in the TME (Steggerda et al., 2017). This agent is also being developed in combination with pembrolizumab (Papadopoulos et al., 2017). The arginase-targeting strategy also extends beyond CB-1158: additional compounds target immunosuppressive Arg1/Arg2 activity, including OAT-1746 and “compound 9,” which restored effector T-cell function *in vitro* but did not produce anti-tumor effects in the same setting (Steggerda et al., 2017; Czystowska-Kuzmicz et al., 2019). More broadly, the arginine–NO axis is also targeted pharmacologically to reduce suppressive myeloid programs and improve T-cell recruitment and priming. Prim-O-glucosylcimifugin (POG) inhibits arginine metabolism in PMN-MDSCs and synergizes with PD-1 blockade (Gehad et al., 2012; Gao W. et al., 2019). In parallel, iNOS inhibition with L-NNA (N(ω)-nitro-L-arginine) restores vascular E-selectin expression and enhances T-cell recruitment (Gehad et al., 2012; Gao W. et al., 2019). Additional NO pathway modulators include NOHA (NG-hydroxy-L-arginine, an arginase-related intermediate/inhibitory modulator) and L-NAME (Nω-nitro-L-arginine methyl ester, an NOS inhibitor), which increase co-stimulatory molecule expression in DCs and support antigen presentation (Simioni et al., 2017). Finally, the arginine axis is also linked to clinical stratification, as circulating L-arginine predicts survival in cancer patients treated with ICIs (Peyraud et al., 2022). Overall, arginine interventions are described as being used either to deprive susceptible tumors of a critical amino acid or to remove arginine-consuming immunosuppression so that T-cell function can be restored, particularly in combination with ICIs.

The tryptophan–kynurenine pathway is a major immunosuppressive pathway in the TME and remains a therapeutic target, but clinical results have shifted the strategy toward combination treatment and multi-node inhibition. A central target in this pathway is IDO1. As single agents, IDO1 inhibitors have shown limited efficacy in clinical trials, including epacadostat, indoximod, and navoximod (Soliman et al., 2016; Beatty et al., 2017; Nayak-Kapoor et al., 2018). This has redirected the pharmacologic focus toward combining tryptophan-pathway inhibitors with checkpoint blockade. For example, epacadostat plus pembrolizumab showed broad anti-tumor activity in ECHO-202/KEYNOTE-037 (Mitchell et al., 2018), but the phase III ECHO-301/KEYNOTE-252 trial in unresectable/metastatic melanoma did not improve progression-free survival compared with pembrolizumab plus placebo (Long et al., 2019). Pharmacologic strategies in this pathway also extend beyond IDO1 by targeting parallel nodes in kynurenine signaling. In particular, kynurenine-mediated immunosuppression is largely linked to AhR. This makes AhR inhibition a relevant therapeutic option, especially in settings where kynurenine production and IDO activity contribute to resistance to anti-PD-1 therapy (Botticelli et al., 2020). Examples of this approach include DMF (3′,4′-dimethoxyflavone), which blocks formation of the nuclear AhR complex, and DMF-based combinations that enhance PD-1 blockade in CD8^+^ T cells when used with carboxyamidotriazole (Lee and Safe, 2000; Shi J. et al., 2019). In parallel, dual IDO1/TDO inhibitors are being developed to reduce pathway redundancy and maintain suppression of kynurenine signaling (Naing et al., 2020; Liang et al., 2021).

A fourth therapeutic area within amino acid metabolism targets oncometabolite-producing enzymes, especially mutant IDH and 2-HG (2-hydroxyglutarate). In this setting, IDH is a clinically actionable target. Ivosidenib (an IDH1 inhibitor) has been evaluated in a phase I trial after allogeneic transplantation, with dose-escalation cohorts and safety/maximum tolerated dose endpoints (NCT03564821) (Shallis and Podoltsev, 2021). It is also being tested in combination with ruxolitinib in advanced Philadelphia chromosome-negative myeloproliferative neoplasms carrying IDH1 mutations (NCT06291987), while enasidenib (an IDH2 inhibitor) has completed phase I/II evaluation in IDH2-mutant hematologic malignancies, including relapsed/refractory acute myeloid leukemia, with outcomes reported in terms of objective response rate and prolonged survival (NCT01915498) (Wang X. et al., 2025). These agents are also being integrated into immunotherapy strategies, including clinical combinations such as olutasidenib + nivolumab (NCT03684811) and ivosidenib + nivolumab (NCT04056910) (Wang Y. et al., 2022). The rationale for these combinations is based on preclinical evidence showing that IDH inhibition can restore T-cell activity and reprogram immunosuppressive myeloid cells, thereby reversing 2-HG-mediated immune suppression (Bunse et al., 2018; Friedrich et al., 2021). Additional mutant-IDH inhibitors, including AGI-5198 (mutant IDH1) and AGI6780 (mutant IDH2), also show anti-cancer activity in glioma and leukemia models, although their effects on T cell-mediated anti-tumor immunity in patients remain unclear (Rohle et al., 2013; Wang et al., 2013). Overall, IDH/2-HG targeting functions not only as a tumor-directed precision approach but also as a potential immunometabolic strategy to improve responsiveness to checkpoint blockade in IDH-mutant cancers.

Two additional amino acid-linked therapeutic axes warrant inclusion. First, the GCN2 kinase, activated downstream of amino acid deprivation (tryptophan, leucine, and arginine) as described in Section 4, represents an actionable immunometabolic checkpoint. GCN2 activation drives T cell arrest via eIF2α-ATF4 signaling and TCR/CD3ζ downregulation, promotes Treg differentiation, and enables MDSC-mediated suppression; pan-cancer analyses confirm that high GCN2 expression correlates with reduced effector T cell signatures and expanded Tregs (Zheng et al., 2023; Chen L. et al., 2024). Selective small-molecule GCN2 inhibitors (including RPT-GCN2i, DA-4507, and HCI-1046) restore CD4^+^/CD8^+^ T cell proliferation and effector function under nutrient stress, reprogram MDSCs toward inflammatory phenotypes, and slow tumor growth in syngeneic models in a CD8^+^ T cell-dependent manner; efficacy is further enhanced in combination with checkpoint blockade or anti-VEGFR therapy (Marshall et al., 2021; Ravishankar et al., 2019; Jackson et al., 2022). Importantly, GCN2 inhibition is context-dependent: in glioma, GCN2 supports CD8^+^ T cell survival under extreme amino acid deprivation, and its inhibition can be detrimental (Rashidi et al., 2020), underscoring the need for tumor-type-specific evaluation. Second, polyamine biosynthesis represents a downstream effector of the arginine-ornithine axis that actively sustains TAM immunosuppression. Tumor-derived arginine fuels arginase-1 and ODC1 activity in TAMs, generating polyamines (putrescine, spermidine, and spermine) that promote M2-like polarization via the polyamine–EIF5A–hypusine axis and epigenetic reprogramming (Zhu et al., 2025; Chia et al., 2022). Polyamine blocking therapy (PBT), combining ODC inhibition (DFMO) with polyamine transport blockade, reduces M2-like TAMs and MDSCs, increases inflammatory CD80^+^/CD11c^+^ macrophages, and markedly enhances anti-PD-1 efficacy in otherwise resistant tumors in preclinical breast cancer and melanoma models (Gilmour et al., 2020; Holbert et al., 2022; Alexander et al., 2017). These two axes (GCN2 and polyamine biosynthesis) extend the therapeutic reach of amino acid metabolism targeting beyond the enzymes already discussed, providing complementary entry points to reverse myeloid and T-cell suppression in amino acid-depleted tumor microenvironments.

Together, these amino acid-directed interventions illustrate how targeting glutamine, arginine, tryptophan–kynurenine signaling, and IDH/2-HG can reshape nutrient availability and immunosuppressive metabolism in the TME, providing a rationale for combination strategies with ICIs; the key approaches, mechanisms, and reported outcomes are summarized in Table 3.

**TABLE 3:** Amino acid metabolism. Summary of pharmacologic agents targeting amino acid metabolism, including combination strategies, metabolic effects, and outcomes.

Drug	Combination therapy	Primary metabolic effect	Outcome	Key references
CB-839 (GLS1 inhibitor)	Anti-PD-1/anti-CTLA-4	Decreases glutaminolysis	Increases effector T-cell infiltration and improved checkpoint activity (mouse melanoma)	Yang W. H. et al. (2021); Varghese et al. (2021), NCT02071862, NCT02771626
Trigriluzole (BHV-4157)	Nivolumab or pembrolizumab	Glutamate deprivation strategy	Decreases glutamate availability	Silk et al. (2020), NCT03229278
DON	—	Broad glutamine-enzyme targeting	Resistance/precision limitations	Lemberg et al. (2018)
ADI-PEG20	Pembrolizumab	Decreases extracellular arginine	Arginine depletion	Zou et al. (2019), NCT03254732
CB-1158/INCB001158	Pembrolizumab	Increases local arginine (Arg1/Arg2 axis)	Restores arginine availability with tumor regression and improved T-cell function; blocks myeloid suppression	Miret et al. (2019), Steggerda et al. (2017), Papadopoulos et al. (2017)
OAT-1746	—	Arg1/Arg2 targeting	Reduced Arg1-mediated suppression	Czystowska-Kuzmicz et al. (2019)
Prim-O-glucosylcimifugin (POG)	PD-1 blockade	Inhibits arginine metabolism in PMN-MDSCs	Synergy with PD-1 blockade	Gehad et al. (2012); Gao W. et al. (2019)
L-NNA (iNOS inhibitor)	—	Decreases NO signaling	Restores E-selectin; increases T-cell recruitment	Gehad et al. (2012), Gao W. et al. (2019)
NOHA/L-NAME	—	NO/arginine pathway modulation	Increases DC co-stimulation; supports antigen presentation	Simioni et al. (2017)
Epacadostat (IDO1)	Pembrolizumab	Decreases Trp catabolism via IDO1	Broad activity but no PFS improvement in melanoma	Mitchell et al. (2018), Long et al. (2019)
Indoximod/navoximod (IDO1)	—	Decreases Trp catabolism via IDO1	Poor efficacy as monotherapy	Soliman et al. (2016), Beatty et al. (2017), Nayak-Kapoor et al. (2018)
Linrodostat (BMS-986205)	Nivolumab + ipilimumab (trials)	IDO-pathway inhibition	Decreases Trp- > Kyn immunosuppression	NCT02658890
GCN2 inhibitors (RPT-GCN2i, DA-4507, HCI-1046)	Checkpoint blockade/anti-VEGFR	Blocks GCN2–eIF2α–ATF4 stress response	Restores CD4^+^/CD8^+^ function, reprograms MDSCs; slows tumor growth; context-dependent (detrimental in glioma)	Marshall et al. (2021), Ravishankar et al. (2019), Jackson et al. (2022), Rashidi et al. (2020)
DFMO + polyamine transport inhibitor (PBT)	Anti-PD-1	ODC inhibition → ↓ polyamine biosynthesis → M2 TAM depolarization	↓ M2 TAMs/MDSCs; ↑ inflammatory macrophages; markedly enhances anti-PD-1 in resistant tumors	Gilmour et al. (2020), Holbert et al. (2022), Alexander et al. (2017)
3′,4′-Dimethoxyflavone (DMF)	Carboxyamidotriazole ± PD-1 blockade	Blocks nuclear AhR complex	Enhanced PD-1 blockade in CD8^+^ T cells	Lee and Safe (2000); Shi J. et al. (2019), Botticelli et al. (2020)
Dual IDO1/TDO inhibitors	—	Reduce pathway redundancy	Broader Trp- > Kyn suppression	Naing et al. (2020), Liang et al. (2021)
Ivosidenib/enasidenib/olutasidenib (IDH inhibitors)	Nivolumab (listed combos)	Decreases 2-HG-linked effects	Restored T-cell activity and myeloid reprogramming	Bunse et al. (2018), Friedrich et al. (2021); NCT03684811; NCT04056910
AGI-5198/AGI6780	—	Mutant IDH inhibition	Anti-cancer potential; immune impact unclear in patients	Rohle et al. (2013), Wang et al. (2013)

### 5.4 Under pressure: tuning AMPK, mTOR, and hypoxia/HIF for therapy

Therapeutic manipulation of nutrient- and oxygen-sensing circuits has become a practical way to modulate both tumor fitness and immune competence in the TME because these pathways sit upstream of broad metabolic programs and can be tuned pharmacologically rather than by targeting single downstream enzymes. Three intervention hubs recur, namely, AMPK, mTOR, and hypoxia/HIF signaling, and each offers druggable entry points with distinct immunometabolic consequences.

AMPK modulation is described as a means to impose or exploit metabolic stress, but the therapeutic objective is not simply “activate AMPK” or “turn it off”; rather, AMPK signaling must be titrated to avoid trading tumor control for impaired effector immunity. High AMPK activity has been associated with the persistence of suppressive populations (TAMs, MDSCs, and Tregs) (Blagih et al., 2015; Siska and Rathmell, 2015; Eichner et al., 2019), and excessive AMPK signaling can restrain effector function, yet the loss of AMPKα in antigen-presenting cells and T cells can heighten inflammatory outputs and glycolysis/IFN-γ production (MacIver et al., 2011; Carroll et al., 2013) while also increasing vulnerability to metabolic stress *in vivo* (Blagih et al., 2015). From a pharmacologic standpoint, metformin is a clinically translatable example of AMPK-directed metabolic modulation. It enters mitochondria via OCT transporters and disrupts mitochondrial complex I coupling, increasing the AMP/ATP ratio and activating the LKB1–AMPK pathway. Downstream, this shift in signaling toward mTOR1 inhibition and mTOR2 activation also inhibits ACC, reducing FAS and lipid accumulation (Cioce et al., 2020). In parallel, metformin is discussed as suppressing glycolytic throughput in cancer cells via the inhibition of PFK1 and via suppression of HIF-1α-induced glycolytic enzymes (Hu L. et al., 2019). Metformin is associated with reduced tumor burden in preclinical models (Anisimov et al., 2005; Bodmer et al., 2010) and with supportive findings in human cohorts, including colorectal cancer (Cheng et al., 2020). It has also been evaluated in clinical settings, including metformin plus annual MRI surveillance in Li-Fraumeni syndrome (NCT04374192) (Dixon-Zegeye et al., 2024) and a phase I dose-escalation study combining metformin with vincristine, irinotecan, and temozolomide (NCT01529593) (Metts et al., 2023). Importantly for immunotherapy design, metformin is also linked to functional “re-oxygenation” of tumors, where metformin-mediated reduction of tumor hypoxia potentiated responsiveness to PD-1 blockade (Scharping et al., 2017). This frames AMPK-directed drugs not only as tumor metabolic modifiers but also as microenvironmental conditioners that can improve ICI performance.

mTORC1/2 signaling is positioned as the central integration point for nutrient sufficiency, growth signals, and immune differentiation, making it a powerful but inherently risk-prone pharmacologic target. mTORC1 activation is promoted by amino acid availability (including leucine/isoleucine) (Saxton and Sabatini, 2017), and mTORC1 supports glycolytic and anabolic programs required for robust effector responses. However, direct inhibition (e.g., rapamycin) has opposing immunologic readouts depending on context. Rapamycin can enhance CD8^+^ memory formation (Araki et al., 2009), yet it is also described as immunosuppressive and capable of abolishing vaccine-combined CD8^+^ anti-tumor responses. Similarly, the Treg compartment responds bidirectionally: excessive mTORC1 can impair suppression, whereas low mTORC1 can stabilize suppressive function (Gerriets et al., 2016; Chapman et al., 2018), and importantly, effective eTreg generation is described as requiring mTORC1, raising concern that chronic pathway suppression may carry autoimmunity risks (Sun et al., 2018). These data collectively argue for selective, schedule-aware, and combination-dependent mTOR targeting, using mTOR modulation to reprogram immune states (e.g., memory-leaning phenotypes) without undermining antigen-driven effector expansion or vaccine synergy.

Therapeutic modulation of oxygen sensing focuses less on the abstract presence of hypoxia and more on druggable consequences: the hypoxic TME is associated with impaired effector differentiation, proliferation, and IFN-γ output (Cho et al., 2016), while TCR activation can induce HIF programs that enable inflammatory glycolysis under hypoxia (Cho et al., 2019; Chen et al., 2020). Because HIF signaling can support both tumor adaptation and immune reprogramming, HIF-targeted interventions are presented as potentially useful but mechanistically nuanced, with reports of divergent effects across cytotoxic T lymphocytes and Tregs via FoxP3-associated regulation and mitochondrial metabolic shifts (Clambey et al., 2012; Gropper et al., 2017; Palazon et al., 2017). A pharmacologically attractive angle is therefore not only direct pathway interference but also TME conditioning that reduces hypoxia and improves immune infiltration/function, illustrated by the metformin–PD-1 interaction noted above. Hypoxia also intersects with suppressive myeloid programming: HIF-1α-dependent arginase activity and NO are linked to the induction of suppressive surface molecules (Chiu et al., 2017; Deng et al., 2019), and hypoxia is described as limiting DC stimulatory capacity while promoting Tregs homing (Winning and Fandrey, 2016), which provides additional rationale for combining oxygen-modulating strategies with immunotherapy. A complementary and increasingly actionable strategy is to pharmacologically pre-condition immune cells, especially adoptively transferred T cells, so they better withstand nutrient limitation and oxygen stress after infusion.

*Ex vivo* metabolic programming is another strategy to improve the function of therapeutic T cells function under nutrient and oxygen stress. Several interventions directly target mitochondrial function and nutrient-sensing pathways, including SS-31 (Bendavia) to reduce mitochondrial calcium stress and improve electron transport chain efficiency (Corrado et al., 2020; Mitchell et al., 2020), enforced PGC1α expression to increase intratumoral metabolic capacity and effector function (Scharping et al., 2016), and mitochondrial pyruvate carrier (MPC) inhibition (genetic deletion or UK5099) to promote a memory-like CD8^+^ T-cell phenotype and prolong CAR-T activity *in vivo* (Wenes et al., 2022). Additional expansion-phase strategies include 2-DG (Sukumar et al., 2013), IL-21 plus LDH inhibition (Hermans et al., 2020), the AKT inhibitor AKTI-1/2 (Crompton et al., 2015), and transient BD98 exposure (a cBAF chromatin-remodeling complex inhibitor) to improve CAR-T efficacy in a solid tumor model (Guo et al., 2022).

Overall, these approaches support the same principle: improving mitochondrial capacity and metabolic fitness before infusion can make adoptive cell therapies more effective in the metabolically hostile TME, partly by modulating glycolysis or glutamine metabolism during *ex vivo* preparation to enhance persistence and effector function. Together with pharmacologic control of AMPK–mTOR and hypoxia/HIF signaling in tumors, this provides a coherent framework to reduce tumor metabolic adaptation while preserving or strengthening immune-cell function under stress, as summarized in Table 4.

**TABLE 4:** Nutrient and oxygen sensing + ex vivo metabolic conditioning. Summary of drug-based interventions targeting nutrient and oxygen sensing pathways, as well as *ex vivo* metabolic conditioning strategies, including combination settings, metabolic effects, and outcomes.

Drug	Combination therapy	Primary metabolic effect	Outcome	Key references
Metformin	PD-1 blockade	AMPK-linked signaling; hypoxia conditioning	Reduction of tumor hypoxia potentiated PD-1 blockade	Scharping et al. (2017)
SS-31 (Bendavia)	ACT/CAR-T (*ex vivo*)	Improves mitochondrial fitness	Improved ACT/CAR-T performance in models	Corrado et al. (2020), Mitchell et al. (2020)
UK5099 (MPC inhibitor)	CAR-T (*ex vivo*)	Memory bias via pyruvate flux modulation	Improved CAR-T performance in models	Wenes et al. (2022)
2-DG (*ex vivo* conditioning)	ACT/CAR-T (*ex vivo*)	Glycolysis modulation during expansion	Improved ACT/CAR-T performance in models	Sukumar et al. (2013)
IL-21 + LDH inhibition	ACT/CAR-T (*ex vivo*)	Expansion-phase metabolic conditioning	Improved ACT/CAR-T performance in models	Hermans et al. (2020)
AktI-1/2	ACT/CAR-T (*ex vivo*)	Expansion-phase metabolic conditioning	Improved ACT/CAR-T performance in models	Crompton et al. (2015)
BD98	CAR-T (*ex vivo*)	Expansion-phase conditioning (cBAF inhibition)	Improved CAR-T efficacy in solid tumor model	Guo et al. (2022)

## 6 Conclusion

Metabolic rewiring is not a secondary adaptation of cancer cells but a central organizing force that shapes tumor progression and immune fate. Across glucose, lipid, and amino acid pathways, tumors reconfigure nutrient fluxes to sustain proliferation, redox balance, biosynthesis, and epigenetic plasticity. In doing so, they remodel the TME into a nutrient-deprived, acidified, lipid-enriched, and hypoxic niche that profoundly alters immune cell function.

This metabolic remodeling generates a dynamic tug-of-war. Effector cells such as NK cells and CD8^+^ T lymphocytes rely on tightly coordinated glycolysis, mitochondrial integrity, and amino acid sufficiency to sustain cytotoxicity. Within the TME, however, glucose restriction, lactate accumulation, arginine and tryptophan depletion, lipid overload, and redox stress progressively destabilize mTOR signaling, mitochondrial fitness, and transcriptional programs, culminating in dysfunction and exhaustion. To better connect metabolic rewiring with clinically relevant immune exhaustion and therapeutic response, the main metabolic alterations in the tumor microenvironment and their effects on immune dysfunction and immunotherapy outcomes are summarized in Table 5.

**TABLE 5:** Metabolic determinants of immune exhaustion and their implications for cancer immunotherapy. Key metabolic alterations in the tumor microenvironment are associated with specific mechanisms of immune dysfunction and exhaustion in antitumor immune cells. These metabolic constraints contribute to immunotherapy resistance but also represent actionable targets for therapeutic intervention aimed at restoring immune fitness and enhancing treatment efficacy.

Metabolic alteration in the TME	Mechanistic impact on immune cells	Exhaustion/dysfunction phenotype	Implications for immunotherapy
Glucose deprivation (tumor glycolysis)	Reduced glycolytic flux, ↓ PEP → impaired Ca^2+^–NFAT signaling; ↓ mTORC1 activity	↓ IFN-γ production, reduced proliferation, impaired cytotoxicity (CD8^+^ T cells, NK cells)	Limits response to ICIs; restoring glucose availability or metabolic fitness enhances anti-PD-1 efficacy
Lactate accumulation/acidosis	Inhibition of glycolysis (↓ NAD^+^/NADH ratio), intracellular acidification; suppression of NFAT signaling	T-cell dysfunction, reduced cytokine production, NK cell inhibition, DC tolerogenic shift	Targeting LDHA/MCTs or buffering acidity improves T-cell function and synergizes with ICIs
Hypoxia/HIF-1α activation	Stabilization of HIF-1α → metabolic shift toward glycolysis; altered differentiation programs	Promotion of Tregs, MDSCs, dysfunctional DCs; impaired effector T-cell responses	Hypoxia-targeting strategies (e.g., metformin, HIF modulation) improve ICI responsiveness
mTOR dysregulation	Impaired nutrient sensing → reduced anabolic metabolism in effector cells	T-cell exhaustion, reduced effector differentiation; persistence of suppressive populations	Fine-tuned mTOR modulation can enhance memory T-cell formation and improve immunotherapy outcomes
Glutamine depletion	↓ c-Myc, impaired nucleotide biosynthesis and redox balance	Reduced T-cell proliferation and activation; impaired NK cell function	Glutamine-targeting therapies can reprogram TME and enhance checkpoint blockade efficacy
Arginine depletion (Arg1 activity)	↓ mTORC1 signaling, impaired TCR signaling (ζ-chain downregulation)	T-cell anergy, reduced proliferation and cytokine production	Arginase inhibitors restore T-cell function and improve response to ICIs
Tryptophan depletion/kynurenine accumulation (IDO axis)	Activation of AhR signaling → transcriptional reprogramming	T-cell exhaustion, Treg expansion, immune tolerance	IDO/AhR targeting combined with ICIs aims to overcome resistance mechanisms
Lipid accumulation/oxidized lipids	ER stress, lipid peroxidation, ferroptosis susceptibility	CD8^+^ T-cell exhaustion, impaired DC antigen presentation	Targeting lipid metabolism (e.g., CD36 and ACAT1) enhances T-cell function and synergizes with ICIs

Conversely, suppressive populations (including TAMs, neutrophils, regulatory T cells, and MDSCs) demonstrate remarkable metabolic plasticity. By integrating glycolytic amplification, FAO, arginine metabolism, and redox buffering systems, these cells adapt to metabolic stress and reinforce immune suppression. Thus, metabolic pressure does not uniformly inhibit immunity; it selectively reshapes it. As summarized in Table 5, metabolic dysfunction within the tumor microenvironment directly translates into clinically relevant immune exhaustion phenotypes that critically influence the efficacy of immunotherapy. Key alterations such as glucose deprivation, lactate accumulation, hypoxia, and amino acid depletion converge on common regulatory nodes, including mTOR signaling, redox balance, and transcriptional control, ultimately impairing effector T and NK cell function while promoting suppressive populations such as regulatory T cells, tumor-associated macrophages, and myeloid-derived suppressor cells. These metabolic constraints not only drive progressive immune dysfunction and exhaustion but also represent major determinants of resistance to immune checkpoint inhibitors. Importantly, therapeutic strategies targeting these metabolic vulnerabilities—including modulation of glycolysis, lactate transport, amino acid metabolism, lipid handling, and nutrient-sensing pathways—have demonstrated the potential to restore immune cell fitness and enhance responsiveness to immunotherapy. Together, these findings reinforce the concept that metabolic rewiring is not only a hallmark of tumor progression but also a critical and actionable interface linking immune escape to therapeutic resistance.

Importantly, tumor and immune metabolism are not parallel systems, but interdependent circuits engaged in constant negotiation. The outcome of this metabolic dialog influences not only tumor growth but also responsiveness to immunotherapy. Metabolism, therefore, emerges as a regulatory interface: one that can be therapeutically tuned to shift the balance from immune evasion toward durable anti-tumor immunity.

## 7 Future perspectives

The next phase of cancer immunometabolism research will require greater spatial, temporal, and cellular resolution. Tumors are metabolically heterogeneous ecosystems, and nutrient gradients vary across regions, disease stages, and therapeutic contexts. Integrating metabolomics, single-cell transcriptomics, spatial profiling, and functional immune analyses will be essential to map how metabolic circuits evolve during progression and treatment.

Therapeutically, the challenge is no longer simply to inhibit tumor metabolism but to recalibrate metabolic ecosystems. Because immune cells share many metabolic dependencies with tumor cells, indiscriminate metabolic blockade risks impairing anti-tumor immunity. Rational combination strategies (pairing tumor-directed metabolic inhibitors with ICB, co-stimulatory agonists, or adoptive cell therapies) offer a more nuanced approach. In parallel, *ex vivo* metabolic conditioning of therapeutic lymphocytes may enhance their resilience within nutrient- and oxygen-restricted environments.

A deeper understanding of metabolic plasticity will also be crucial. Many suppressive immune populations thrive precisely because they exploit alternative fuel sources. Identifying context-specific metabolic vulnerabilities, rather than broadly targeting entire pathways, may allow selective disruption of tumor-promoting circuits while preserving or enhancing effector function.

Ultimately, metabolic pathway rewiring should be viewed not merely as a hallmark of cancer but as a strategic battlefield. By learning how to manipulate the rules of metabolic engagement, future therapies may transform the tumor–immune tug-of-war into a coordinated and sustained anti-tumor response.

## Glossary

2-DG: 2-Deoxy-D-glucose
2-HG: 2-Hydroxyglutarate
3PO: 3-(3-pyridinyl)-1-(4-pyridinyl)-2-propen-1-one (PFKFB3 inhibitor)
4-1BB: CD137 co-stimulatory receptor (TNF receptor superfamily)
A2B: Adenosine receptor A2B
ABCA1/ABCG1: ATP-binding cassette transporters A1 and G1
ACC1: Acetyl-CoA carboxylase 1
ACAT1: Acetyl-CoA acetyltransferase 1
Acod1: Aconitate decarboxylase 1
AhR: Aryl hydrocarbon receptor
α-KG: α-Ketoglutarate
APOE: Apolipoprotein E
Arg1/Arg2: Arginase 1/arginase 2
ASCT2: Alanine, serine, cysteine transporter 2 (SLC1A5)
ATF4: Activating transcription factor 4
BCAT1: Branched-chain amino acid transaminase 1
BCKA: Branched-chain keto acid
CAT-1/CAT-2B: Cationic amino acid transporter 1/2B
BNBZ: Benitrobenrazide
CCL4/CCL5: C-C motif chemokine ligand 4/5
cBAF: Canonical BRG1/BRM-associated factor (chromatin remodeling complex)
cDC1: Conventional dendritic cell type 1
CCR2/CCR7: C-C motif chemokine receptor 2/7
cGAS–STING: cyclic GMP-AMP synthase–stimulator of interferon genes
CD36: Cluster of differentiation 36 (fatty acid translocase)
COX-2: Cyclooxygenase-2
CLEC9A: C-Type lectin domain family 9 member A
CTLA-4: Cytotoxic T-lymphocyte-associated protein 4
CPT1α/CPT2: Carnitine palmitoyltransferase 1α/2
CXCR2/3: C-X-C motif chemokine receptor 2/3
CXCL1/2/5/8/9/10: C-X-C motif chemokine ligands 1, 2, 5, 8, 9, 10
DFMO: Difluoromethylornithine (ODC inhibitor)
DLST: Dihydrolipoamide S-succinyltransferase
DMF: 3′,4′-Dimethoxyflavone
DON: 6-Diazo-5-oxo-L-norleucine
EIF5A: Eukaryotic initiation factor 5A
ERBB2: Erb-B2 receptor tyrosine kinase 2 (HER2)
FABP5: Fatty acid binding protein 5
FAS/FASL: Fas receptor/Fas ligand
FATP2: Fatty acid transport protein 2 (SLC27A2)
FBP1: Fructose-1,6-bisphosphatase 1
FLCN: Folliculin
FLT3L: FMS-like tyrosine kinase 3 ligand
FoxP3/FOXP3: Forkhead Box P3 (Treg master transcription factor)
G6PD: Glucose-6-phosphate dehydrogenase
GAPDH: Glyceraldehyde-3-phosphate dehydrogenase
GCN2: General control non-derepressible 2 (kinase)
GITR: Glucocorticoid-induced TNFR-related protein
GLS/GLS1: Glutaminase/glutaminase 1
GLUD: Glutamate dehydrogenase
GLUT1/GLUT3: Glucose transporter 1/3
GM-CSF: Granulocyte–macrophage colony-stimulating factor
GOx: Glucose oxidase
GPR37/GPR81: G Protein-coupled receptor 37/81
GPX4: Glutathione peroxidase 4
GZMB: Granzyme B
H3K18la: Histone H3 lysine 18 lactylation
H3K27/H3K79: Histone H3 lysine 27/79 methylation marks
HBP: Hexosamine biosynthesis pathway
HER2: Human epidermal growth factor receptor 2 (ERBB2)
HIF-1α: Hypoxia-inducible factor 1-alpha
HK2: Hexokinase 2
HSP70: Heat shock protein 70
ICOS: Inducible T-cell co-stimulator
IDH/IDH1/IDH2: Isocitrate dehydrogenase/isoforms 1 and 2
IDO/IDO1: Indoleamine 2,3-dioxygenase/isoform 1
iNOS/NOS2: Inducible nitric oxide synthase
IRE1: Inositol-requiring enzyme 1
JMJD3: Jumonji domain-containing protein 3 (KDM6B)
LAG-3: Lymphocyte-activation gene 3
LAT1: Large neutral amino acid transporter 1 (SLC7A5/SLC3A2)
LCK: Lymphocyte-specific protein tyrosine kinase
LDH/LDHA: Lactate dehydrogenase/isoform A
LKB1: Liver kinase B1 (STK11)
LOx: Lactate oxidase
L-NAME: Nω-nitro-L-arginine methyl ester (NOS inhibitor)
L-NNA: N(ω)-nitro-L-arginine (iNOS inhibitor)
MCAD: Medium-chain acyl-CoA dehydrogenase
MCT1/MCT4: Monocarboxylate transporter 1/4
MEF2C: Myocyte enhancer factor 2C
MGLL: Monoacylglycerol lipase
MPC: Mitochondrial pyruvate carrier
NFAT: Nuclear factor of activated T-cells
NKG2D: Natural killer group 2 member D (activating receptor)
NOHA: NG-hydroxy-L-arginine
NRF2: Nuclear factor erythroid 2-related factor 2
ODC1: Ornithine decarboxylase 1
PAD4: Peptidyl arginine deiminase 4
OX40: CD134 co-stimulatory receptor (TNF receptor superfamily)
PBT: Polyamine blocking therapy
PD-1/PD-L1: Programmed cell death protein 1/programmed death-ligand 1
PFK1: 6-Phosphofructokinase 1
PFKFB3: 6-Phosphofructo-2-kinase/fructose-2,6-bisphosphatase 3
PGC1α: Peroxisome proliferator-activated receptor gamma coactivator 1-alpha
PGE2: Prostaglandin E2
PHGDH: Phosphoglycerate dehydrogenase
PKM/PKM2: Pyruvate kinase M1/2/isoform 2
PMN-MDSC: Polymorphonuclear MDSC
POG: Prim-O-glucosylcimifugin
PP2A: Protein phosphatase 2A
PPARα/PPARδ/PPARγ: Peroxisome proliferator-activated receptor α/δ/γ
PSGL-1: P-Selectin glycoprotein ligand-1
RB1: Retinoblastoma protein 1
SAM: S-adenosylmethionine
SIX1: SIX homeobox 1
SLC1A5: Solute carrier family 1 member 5 (ASCT2)
SLC6A14: Solute carrier family 6 member 14 (amino acid transporter)
SLC7A5: Solute carrier family 7 member 5 (LAT1)
SLC7A11/xCT: Solute carrier family 7 member 11 (cystine/glutamate antiporter)
SLC27A1: Solute carrier family 27 member 1 (fatty acid transporter)
SLC38A1/2/3/5: Solute carrier family 38 members 1, 2, 3, 5 (glutamine transporters)
SLC43A2: Solute carrier family 43 member 2 (methionine transporter)
SR-A/SR-BI: Scavenger receptor class A/class B type I
SREBP1/SREBP2: Sterol regulatory element-binding protein 1/2
TDO: Tryptophan-2,3-dioxygenase
TFEB: Transcription factor EB
Tim-3: T-Cell immunoglobulin and mucin domain-containing protein 3
TRIF: TIR-domain-containing adapter-inducing interferon-β
UDP-GlcNAc: Uridine diphosphate N-acetylglucosamine
VISTA: V-domain Ig suppressor of T-cell activation
XBP1: X-box binding protein 1
XCL1: X-C motif chemokine ligand 1
ZAP-70: Zeta-chain-associated protein kinase 70
